# Denoising DNA deep sequencing data—high-throughput sequencing errors and their correction

**DOI:** 10.1093/bib/bbv029

**Published:** 2015-05-29

**Authors:** David Laehnemann, Arndt Borkhardt, Alice Carolyn McHardy

**Keywords:** next-generation sequencing, high-throughput sequencing, error profile, error correction, error model, bias

## Abstract

Characterizing the errors generated by common high-throughput sequencing platforms and telling true genetic variation from technical artefacts are two interdependent steps, essential to many analyses such as single nucleotide variant calling, haplotype inference, sequence assembly and evolutionary studies. Both random and systematic errors can show a specific occurrence profile for each of the six prominent sequencing platforms surveyed here: 454 pyrosequencing, Complete Genomics DNA nanoball sequencing, Illumina sequencing by synthesis, Ion Torrent semiconductor sequencing, Pacific Biosciences single-molecule real-time sequencing and Oxford Nanopore sequencing. There is a large variety of programs available for error removal in sequencing read data, which differ in the error models and statistical techniques they use, the features of the data they analyse, the parameters they determine from them and the data structures and algorithms they use. We highlight the assumptions they make and for which data types these hold, providing guidance which tools to consider for benchmarking with regard to the data properties. While no benchmarking results are included here, such specific benchmarks would greatly inform tool choices and future software development. The development of stand-alone error correctors, as well as single nucleotide variant and haplotype callers, could also benefit from using more of the knowledge about error profiles and from (re)combining ideas from the existing approaches presented here.

## Sequencing platforms and their errors

We begin with a survey of the errors generated during sequencing by five commonly used high-throughput sequencing platforms: the GS FLX and the GS Junior by 454 [1], the Complete Genomics platform [2], the HiSeq and the MiSeq by Illumina [3], the Personal Genome Machine (PGM) by Ion Torrent [4, 5] and the Real-time Sequencer (RS) by Pacific Biosciences [6]. Further, we include a quick summary of what is published about the very recent MinION platform by Oxford Nanopore [7] that has not been released to general public, yet. A detailed review of the underlying technologies and further platforms is available elsewhere [8, 9]. For all platforms except for the MinION, independent error-assessments exist, but only few studies have systematically compared several platforms [10–13]—with none covering more than four. Also, the analyses vary in focus, reporting on only some of the well-known error types: insertions and deletions (often subsumed as indels), substitutions and coverage biases, such as reduced coverage of certain regions. To determine whether errors are introduced before library preparation (e.g. during pre-amplification steps), during library preparation and amplification or in the sequencing run, comparative experiments under different experimental conditions are required. Such time- and cost-intensive analyses have rarely been performed, and therefore, such distinctions have been made in few instances only [13]. Some properties of nucleic acid sequences are known to raise the error rates for all or most technologies, such as extremes in GC content, long homopolymer stretches, the presence of human promoter sequences and the well-known decay of the base signal along each read. After discussing the error profiles of the individual platforms, we conclude the first part of the review with a direct comparison of all platforms with regard to these aspects.

### 454 pyrosequencing

For 454 pyrosequencers, an overall error rate for the GS FLX [14] and the GS Junior [12] machines has been reported and the indel rate assessed for the GS Junior [10]. All three studies only investigated sequences of intermediate GC content. Nevertheless, their reported error rates (Table 1) support the well-known consensus that—with this technology—indel errors occur an order of magnitude more often than substitution errors. This higher indel error rate is mostly owing to occurrences of homopolymers, i.e. multiple consecutive appearances of the same nucleotide. The distributions of light intensities of individual base flow cycles in the sequencing reaction increasingly overlap for longer homopolymer lengths, leading to insertion and deletion errors in the base calling [18]. Owing to this phenomenon, homopolymers have a higher overall indel error frequency than other sequence stretches [10, 14] and the indel error frequency increases with homopolymer length [10].

**Table 1.** Error rates of high-throughput sequencing platforms (per 100 sequenced bases)

Platform	Subs	SD Subs	Indels	SD Indels	^a^All	SD All
454 GS FLX	0.09000	^b^N/A	0.90000	^b^N/A	0.99000	^b^N/A
454 GS Junior	0.05430	^b^N/A	0.39055	^b^N/A	0.45540	^b^N/A
Complete Genomics	2.30000	^b^N/A	0.01900	^b^N/A	2.31900	^b^N/A
Illumina HiSeq	**0.26400**	0.11238	**0.02561**	0.02351	**0.28467**	0.11875
Illumina MiSeq	**0.24551**	0.11079	**0.00905**	0.01436	**0.29652**	0.18867
Ion Torrent PGM	**0.16985**	0.17253	^c^ **1.45793**	^c^ 1.21924	^c^ **1.63112**	^c^ 1.24217
Pacific Biosciences RS	**1.10286**	0.44761	**15.56571**	3.29386	**16.19250**	3.16667

Data from [10–17]. For all used values from these studies, please see the comprehensive Supplementary Table S1.

Only numbers based on a sufficiently large sample size to provide a standard deviation are set in bold.

Subs = substitution errors per 100 bases; Indels = insertion and deletion errors per 100 bases; SD = standard deviation

^a^Some studies contain only aggregated measures for indels and/or total error. Therefore, the value of All is not necessarily the sum of Subs and Indels.

^b^For these platforms only one sample was available. Therefore, error rates should be considered with care as SDs are not available.

^c^One study with three samples (out of 12 samples in total) used indel-tolerant mapping, resulting in almost 100% of reads being mapped, but also producing much higher indel error rates. This sample also explains the high SD.

454 sequencing data also contains a considerable amount of ambiguous base calls (some callers then output an ‘N’), though at a frequency considerably lower than that of indels and comparable with that of mismatches [14]. Towards the end of the read, ambiguous base calls increase significantly in frequency, as do substitution errors, whereas indel errors show only a slighter but noticeable increase [14, 18]. After a certain point in the read, depending on the machine and chemistry in use, the GC content (as averaged over all reads) also drops drastically, indicating a strong GC bias in later flow cycles (Figure 1 in [19]). At the same time, longer reads surprisingly have a lower average error rate. Gilles *et al.* [14] suggest that reads either have a consistently low or a consistently high error rate, i.e. shorter reads are high error reads that have been trimmed heavily to remove errors towards the end, but the remaining parts still contain more errors on average than the higher quality reads that did not have to be trimmed as much. Finally, an inverse pattern of insertion and deletion calls per well are found across the technology’s picotitre plates: insertions are found more often than deletions in certain parts of the sequencing plates, whereas the effect is inverted for other areas. Contiguous areas of the sequencing plate are thus either enriched for deletions or for insertions, but these patterns do not seem to be consistent between distinct sequencing plates or runs (Figure 3 and Additional File 4 in [14]).

**Figure 1.**
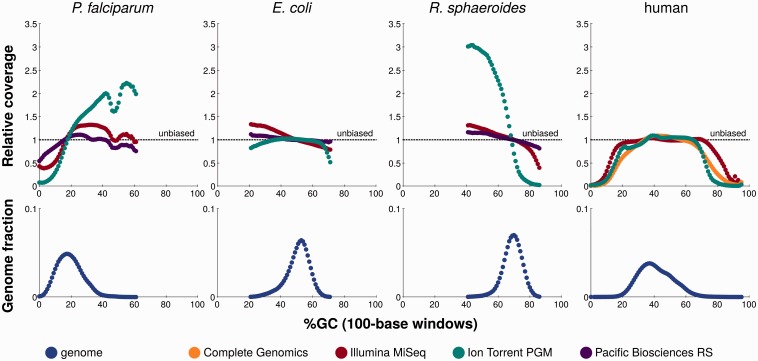
Sequencing coverage across different local GC contents in three microbes (*P. falciparum*, *E. coli* and *R. sphaeroides*) and a human genome. The bottom panels show the relative fraction of 100-base windows in the respective genome having a certain GC content. The top panels show the relative sequencing coverage for 100-base windows with a certain GC content compared to the respective platform sample's average. This figure is aggregated and adapted from Figures 2 and 3 in [13], according to the Creative Commons Attribution license CC-BY 2.0 (http://creativecommons.org/licenses/by/2.0/). A colour version of this figure is available at BIB online: http://bib.oxfordjournals.org.

### Complete Genomics DNA nanoball sequencing

**Figure 2.**
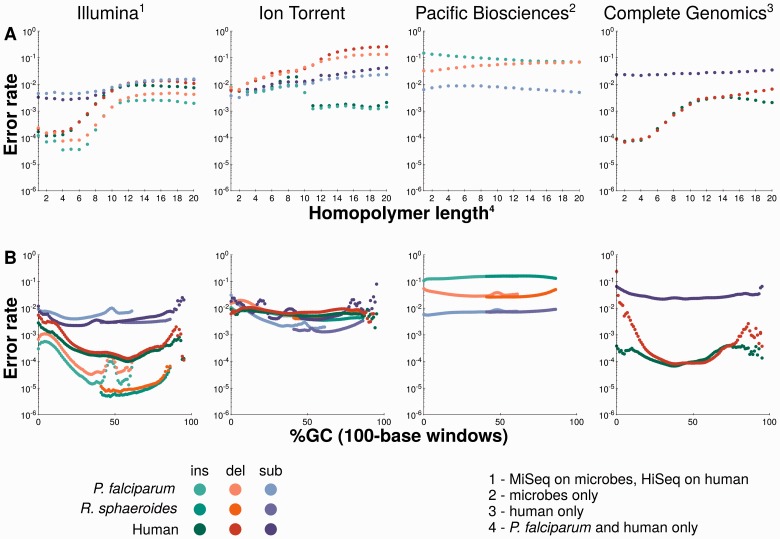
Error rate biases in homopolymers of varying lengths and due to different local GC sequence content. (**A**) Top panels show the average error rates at homopolymers of different lengths per genome and platform. (**B**) Bottom panels show error rates across different GC sequence contents of 100-base windows. This figure is aggregated and adapted from Figures 4 and 5 in [13], according to the Creative Commons Attribution license CC-BY 2.0 (http://creativecommons.org/licenses/by/2.0/). A colour version of this figure is available at BIB online: http://bib.oxfordjournals.org.

Error information for Complete Genomics DNA nanoball sequencing comes from a systematic comparison of platforms on a human genome sample [13]. With this limitation in mind, the reported error rates (Table 1) indicate that substitution errors are two orders of magnitude more common with this technology than are indel errors. The overall error rates were found to be consistent across a wide range of GC sequence contents, apart from the deletion rate that was much higher for sequences with high or low GC content (Figure 2B). These two GC content extremes are also associated with substantially lower read coverage (Figure 1). Finally, indel error rates rise markedly with increasing homopolymer length (Figure 2A; [13]).

**Figure 3.**
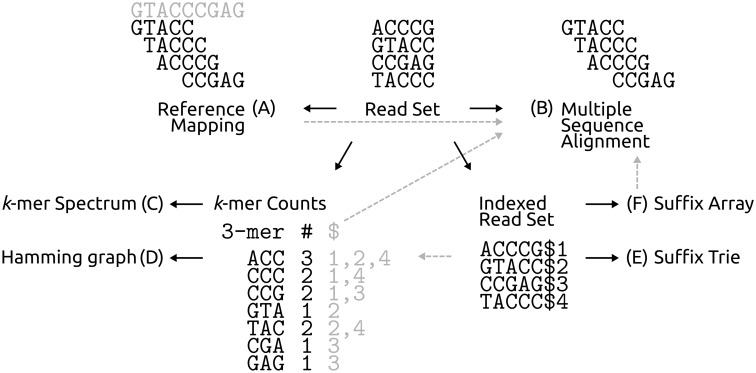
Overview how to generate a pileup from a read set depending on the error correction strategy. (**A**) If a good and close reference is known, reads can be mapped to it. Otherwise, one of the other approaches is necessary: (**B**) A MSA of reads can be formed from pairwise alignments of all read pairs, of all reads with an overlap in an initial mapping to an available reference (dashed grey arrow from A to B), of all reads sharing part of a suffix (dashed grey arrow from F to B) or of all read pairs sharing a *k*-mer seed, identified by a table recording all reads that each *k*-mer occurs in (grey table and respective dashed grey arrows). Also, a simple recording of the count of all *k*-mers can be used to derive (**C**) a *k*-mer Spectrum or (**D**) a Hamming graph (Figure 4), and read suffixes of reads augmented with unique symbol ($*x*) can be used to construct (**E**) a suffix trie (Figure 5) or (F) a suffix array (Figure 6).

### Illumina sequencing by synthesis

The error profiles of the current Illumina sequencing by synthesis platforms HiSeq and MiSeq have been characterized in substantial detail, also drawing on the rigorous analyses of earlier Illumina platforms such as the 1 G [20, 21] and the Genome Analyzer II [22]. Indel errors are an order of magnitude less frequent than substitution errors and Illumina's overall error rate is the lowest of all the technologies (Table 1). For HiSeq, it has been shown that deletions are more common than insertions [15], whereas insertions are more common than deletions in MiSeq data [23]. Substitution errors show a bias towards certain substitutions: A <-> C and G <-> T transversions are by far the most common, each making up around 30 and 25% of all substitutions in HiSeq data [15]. A similar effect has been proven for MiSeq data [23]. Overall, the error rate increases towards the end of reads [15, 23], an issue that can be mostly dealt with by trimming low-quality ends, and is significantly higher in the second read when doing paired end sequencing on the MiSeq [23].

In the Illumina sequencers, modified nucleotides are flushed over a flowcell in cycles. Flowcell tiles containing millions of sequence clusters—each performing individual sequencing reactions—are arranged in lanes along each flowcell. Tiles at both ends of a lane tend to have some cycles with an elevated average error rate. This leads to spatially clustered and flow cycle-specific errors [15]—a phenomenon that might explain the substantial variation in error patterns between different sequencing runs with the same sequencing library, as it is very unlikely that the same templates will be located at border tiles in two different runs [13]. However, further variation can be observed between sequences generated from different libraries prepared from the same material [13], demonstrating that errors are already generated in the preparation steps. Especially the library preparation method and the sequencing primers have been shown to introduce error biases [23]. Also, some errors have been linked to sequence motifs: especially the indel error rate increases after long homopolymer stretches (Figure 2A), in GC-rich sequences (Figure 2B; GGCGGG is the most prominent motif) and around inverted repeats [11, 13, 22]. Fortunately, most of these error rate increases are consistently reflected in lower quality scores for the respective read positions [15]—however, a general overestimation of base calling quality in Illumina data has been reported (e.g. Supplementary Figure S1 in [21]) and any errors introduced in library or sample preparation [e.g. owing to polymerase chain reaction (PCR) amplification] would not be reflected in the sequencing quality scores [23]. But in general, this supports the practice of quality trimming for errors that occur and accumulate at both ends of reads [15] and calls for further use of the quality scores in all downstream analyses. In addition, some sequence-specific errors are strand-specific, which should also help discerning them from genuine polymorphisms in a sample [11, 15, 23]. Finally, the Illumina platforms show a clear drop of read coverage at both extremely high and low GC sequence contents (Figure 1), an effect that PCR-free library construction is able to reduce, but not to eliminate completely [13].

### Ion Torrent semiconductor sequencing

For Ion Torrent's current semiconductor sequencing platform, the PGM, errors have been assessed in detail. Here, indel errors are an order of magnitude more frequent than substitution errors and the overall error rate is substantially higher than in the 454 and Illumina platforms (Table 1). The indel error rate—when measured against a known reference genome—becomes even higher if reads are mapped with indel tolerance: substantially more reads are mapped and more of the actual indels are counted [16]. Such methodological differences between studies explain the high standard deviation of the error rates reported in Table 1.

A substantial fraction of indel errors are caused by homopolymers [10], with the base-calling accuracy decreasing with homopolymer length [16]. While insertion errors are more likely than deletion errors in PGM data in general, deletion errors are more likely for homopolymers overall and increasingly likely, the longer a homopolymer gets [16]—leading to an average under-reporting of homopolymer lengths (Figure 2A; [13]). This is especially pronounced for homopolymer stretches of more than ∼8 nucleotides and may eventually lead to a complete loss of read coverage for homopolymers of more than 14 nucleotides [11]. Another portion of indel errors occurs at very high frequencies at certain positions of the reference genome [16]: these are mostly either A or T insertions or C or G deletions; around 80% of them are run-specific (i.e. requiring sequencing replicates to identify them); and around 7% of them show a significant strand-specificity. Such strand-specific errors have also previously been reported: some connected to homopolymer indels [10], others with no apparent sequence motif connected to them [11].

GC bias has a pronounced effect in PGM sequencing. While the higher overall indel error rate is more stable across extreme GC sequence contents than in Illumina and Complete Genomics sequencing (Figure 2B), sequence coverage is not (Figure 1): it drastically drops at both high and low GC sequence content, both within the human genome and across microbial genomes of different GC sequence content [13]. This is further confirmed by two other studies that demonstrated a strong coverage bias in the very low GC genome of *Plasmodium falciparum* [11] and the complete failure of library preparation for the very high GC content genome of *Deinococcus maricopensis* alongside an elevated error rate for a low GC content genome of *Sulfolobus tokodaii* [16]. It has therefore been suggested that PGM library preparation is only safe for species with (local) GC sequence contents ranging from approximately 20–80% [16].

Finally, error rates on average increase towards the end of reads (Figure 2A; [10, 16]), with a clear periodicity of error rates, which is connected to the Samba flow rhythm [16]: nucleotides are flushed over the sequencing chip separately and certain flows—mostly A and T flows—have significant over-call or under-call rates, which directly connect to insertion and deletion errors, respectively. A Samba flow-specific error model might allow better error correction [16].

### Pacific Biosciences single-molecule real-time sequencing

Even though the reported error rates (Table 1) are supported by more samples than those for 454 pyrosequencing and Complete Genomics, the error profile of the Pacific Biosciences Real-time Sequencer (PacBio RS) is not very well characterized by independent studies, especially regarding PacBio's more recent chemistries. The overall error rate of the earlier chemistries is approximately one order of magnitude larger than that of the Ion Torrent PGM and approximately two orders of magnitude larger than that of the Illumina platforms (Table 1). Within the platform, indel errors are around 15 times more common than substitution errors. The drawback of extremely high error rates—and lower overall throughput, which makes it unaffordable for the amount of DNA in larger genomes such as the human genome [13] or in metagenome studies—is partly offset by two factors: Firstly, very long reads (∼10 kb) make the platform useful for scaffolding of *de novo* sequence assemblies of smaller genomes that also use read data from another platform [11]. Secondly, coverage drops only very slightly at extreme GC sequence content, making this the platform with the lowest GC bias (Figure 1; [13]). With recent increases in data quality and read length, *de novo* assemblies of bacterial genomes from PacBio data alone have become possible [24]. Also, two methodological approaches for the reduction of the error rate have been suggested by the platform vendor: (i) The SMRTbell template, which is an effectively circular, double-stranded DNA template with loops at both ends, enables multiple consecutive readings of the same template. These are then aggregated into a consensus read with a much lower error rate [25]. (ii) Alternatively, redundant coverage from distinct templates of variable sizes can be used to create a correct consensus. In this approach, the more abundant shorter reads provide the coverage redundancy, while the longer reads assure assembly contiguity [26]. Such extra redundancy—through SMRTbell or increased overall coverage—has been independently shown to decrease the overall error rate by an order of magnitude to 1.3 and 2.5%, respectively ([27, 28]; section ‘Platform-specific error correction’).

Furthermore, the error rate was found to be consistent and random for the longest part of each read, deteriorating only slightly for longer reads [17]. The error rate is also unbiased by longer homopolymer stretches—with only a slight increase of deletions and a slight decrease of insertions (Figure 2A)—and across the entire range of GC sequence content (Figure 2B; [13]).

### Oxford Nanopore sequencing

Like PacBio's real-time sequencing, Oxford Nanopore's MinION promises to generate longer reads that will enable better resolution of structural variants and genomic repeat content. However, this technology is even younger than the PacBio RS and all published studies are based on data from the platform developers' MinION Access Programme [29] and are only based on short fragments of DNA (phage or bacterial genomes or single human genes). In addition, the chemistry is under rapid development, constantly improving data quality [30]. The here presented values should thus be taken with care and are neither included in Table 1 nor in the platform comparison below.

Similar to circular consensus sequencing (CCS) reads in PacBio sequencing, a DNA hairpin loop is ligated to the end of a double-stranded DNA molecule, linking the two strands. However, this is only done at one end of the double strand and the MinION thus sequences each strand only once [31]. This results in a ‘template’ read before the hairpin and a (reverse) ‘complement’ read after the hairpin. Wherever possible, a consensus of both is created, a ‘two-dimensional’ (2D) read [31]. Most reported error rates refer only to these 2D reads that represent about 40 to 50% of sequenced bases for the newest published flow cell version R7.3 [30–32] and among those only to the 63 [32] to 90% [30] of reads that map to the respective reference. With such reads, Ammar *et al.* report 7% substitution errors, 13.3% deletion errors and 5.3% insertion errors. Jain *et al.* further narrow their choice of reads down to those marked as high quality by the vendor's base-caller, finally analysing around 24% of the total sequenced nucleotides. Averaging over three replicates of the phage M13, they report 5.1% substitution errors, 7.8% deletion errors and 4.9% insertion errors and also apply their error estimation to the *Escherichia coli* data of Quick *et al.*, where they find 5.3% substitution errors, 9.1% deletion errors and 6.0% insertion errors.

Altogether, these studies roughly agree that substitution and insertion errors occur at a similar rate, while deletion errors are about two times as common. The joint error rate of about 20–25% still clearly exceeds that of PacBio, the other current single-molecule sequencing technique. Also in contrast to PacBio, errors seem to be biased: with substitution errors, A to T and T to A errors are much less likely than all other substitution errors [30] and homopolymer runs seem to increase insertion and deletion error rates [30, 33].

### Platform comparison: errors in connection with GC biases, homopolymers and human promoter sequences

On four of the five available platforms (excluding Oxford Nanopore), sequences with GC content extremes are known to suffer from reduced coverage in the produced reads, with some regions covered by no reads in all platforms except for the PacBio RS (Figure 1; [13, 19]). For 454 pyrosequencing with the GS FLX platform, a drastic decrease in average GC content after a certain read length is a known GC bias [19]. The other four platforms were compared systematically for GC biases by Ross *et al.* [13]: all platforms represent sequences with intermediate GC content consistently and show a decreased coverage of both high and low GC content sequences (Figure 1). PacBio RS gives the most consistent coverage, even at extreme GC sequence contents, but currently does not yet support the sequencing of large genomes (e.g. human) at a reasonable cost. In practice, it therefore only outperforms all other platforms with respect to GC content bias for the analysis of microbial genomes. The Ion Torrent PGM consistently shows the strongest loss of coverage for low and high GC sequence content regions, across both microbial and human genomes. In a low GC content genome, up to 30% of the genome were not covered at all [11], and for a high GC content genome, library preparation failed altogether [16]. Complete Genomics—a platform specifically targeted at human genome sequencing—performs similar to the PGM on the human genome. Here, Illumina's HiSeq platform outperforms both Ion Torrent PGM and Complete Genomics [13]. The coverage bias of these technologies for extreme GC content sequences also could not be amended by combining data from different platforms [13], possibly because the bias profiles are not complementary but rather qualitatively similar, with differences only in the strength of the GC bias.

In addition, several platforms show correlations between GC content or GC-motifs and different kinds of single nucleotide errors [13]. The indel error rates of the PGM were shown to be stable across the GC range (at a high overall level), as were the even higher indel error rates of the PacBio RS. Complete Genomics and Illumina had much lower overall indel error rates, but showed a clear elevation of the deletion error rate at extremely high and extremely low GC contents, an elevation also seen for insertion errors in Illumina data (Figure 2B; [13]). Ion Torrent's PGM is the only platform with a clear elevation of the substitution error rate, especially at very low GC sequence content, while Complete Genomics, Illumina HiSeq and MiSeq and PacBio RS have a mostly stable substitution error rate across the whole range of GC sequence contents, with only very slight elevations at the extremes (Figure 2B). In addition, certain strand-specific and cycle-specific errors in Illumina data have been attributed to GC-rich sequences [11].

With increasing length of homopolymers, all platforms show an increase in the insertion error rate, the deletion error rate or both, except for the PacBio RS with its consistently very high indel error rate (Figure 2A; [13, 14]). Most sensitive to homopolymers is the Ion Torrent PGM, which was found in one study to not produce any reads for homopolymers longer than 14 nucleotides [11].

Inspired by anecdotal evidence, [13] also examined the coverage of human promoter sequences and identified a substantial number of such promoters that are extremely under-covered by all the tested platforms (i.e. Complete Genomics, Illumina HiSeq and Ion Torrent PGM), an effect neither explained by GC sequence content nor by homopolymers. Illumina showed the highest coverage, and thus best performance, when used with improved reagents. However, further more detailed studies of this phenomenon are required. Interestingly, for Illumina's older platform Genome Analyzer II, certain errors have been shown to be associated with inverted repeats [22] and the human genome is known to contain a substantial number of bidirectional promoters [34] for adjacent and oppositely directed genes. These bidirectional promoters are known to have complementary and symmetric base sequence content around their midpoint and might harbour inverted repeats in the form of inversely oriented transcription factor binding sites [35], a connection that could easily be tested with existing data.

## Error correction of read data

We now provide a methodological overview of high-throughput sequencing error correction software that takes the read data as input and outputs corrected reads. We focus on tools where this process can be run as a stand-alone and where a publication or the documentation provides details of the methodology (Tables 3 and 4, Supplementary Table S2). However, we also list software where error correction is only one step of many, as in sequence assemblers and the haplotype inference tools ShoRAH and KEC, especially if they have contributed important error correction ideas or if the error correction tool can be run independently. Initially, we outline the computational methods, i.e. which data structures are used, and how algorithms generate and manipulate them for error detection. Afterwards, we discuss the employed error models, including which features they characterize them by, and the statistical methods, including the utilized models and their parameters. Importantly, we highlight the assumptions made by the different tools and indicate which ones are applicable to which types of data, to facilitate the choice of tools for context specific benchmarks. There are three main assumptions that most high-throughput sequencing error correction approaches make: Firstly, errors (per position) are considered rare compared with correct base calls, given sufficient coverage. Secondly, coverage is seen as uniform across the queried sequence. And thirdly, substitution and indel errors are all thought to be introduced with similar probability at every sequence template position. These assumptions might be reasonable enough, if only overall error rates are known for a certain data type. However, considering that there are also systematic errors (biases) that affect both coverage and error frequencies, more sophisticated approaches allow to more adequately detect and remove errors from sequence data. We begin by describing approaches that are based on these three assumptions, and later discuss error correction approaches that take such effects into consideration.

## Piling up reads over query sequence positions: determining base frequencies by read alignments, *k*-mer counting or read suffixes

To leverage the high coverage—attainable with high-throughput sequencing—for error correction, sequence reads have to be sorted according to the location of the underlying sequence from which they originate. This can be achieved using a variety of techniques, depending on whether a reference sequence is already available, and results in a base pileup or an alignment column. Then, positions with multiple divergent base calls are inspected, to distinguish genuine polymorphisms from sequencing errors—making use of the assumptions that errors are rare and random. The initial step of determining base frequencies over sequence positions is either done by aligning reads or by inspecting the frequency of all substrings of all reads of a defined length *k* (*k*-mers), where the latter can be done using some form of either a *k*-mer counting table or a suffix array. This classification into alignment or *k*-mer-based approaches was adapted from [36].

### Read alignments: Reference mapping and multiple sequence alignment

Wherever a reference genome is available, the base pileup over each sequence position can be done by finding an optimal mapping of each read onto that reference sequence (Figure 3A). However, often no (good) reference genome is available or (substantial) divergence from the reference is expected, which would lead to a reference bias in the alignment and thus in the positional pileup. In this case, reference-free assembly or read grouping strategies can be used to generate a multiple sequence alignment (MSA) of reads, thus generating the positional pileup.

Historically, such reference-free approaches were initially developed for the assembly of Sanger sequencing data, where the few produced reads were longer and coverage was lower. With such small data sets, a pairwise alignment of all read pairs (e.g. [37]) was feasible, and from these pairwise alignments, an MSA (Figure 3B) could easily be constructed and refined [38]. With the larger data sets from the next generation of sequencing platforms, the initial step of all possible pairwise alignments became computationally intractable. Instead, newer approaches first determine read pairs sharing subsequences and then only align those pairs, thus substantially reducing the number of alignments performed. To this end, they borrow from one of the other three pileup approaches we review below: by creating an index table that contains all read substrings of a specified length *k*—so-called *k*-mers (section ‘*k*-mer frequencies and spectrum’)—and recording in which reads they occur (grey read index table in Figure 3; [39, 40]), by doing a reference mapping (grey dashed arrow from A to B in Figure 3; [41, 42]) or by constructing a suffix array (section ‘Suffix tries and arrays, the Burrows Wheeler transform and the Full-text index in Minute space’) of all reads [43] or derivatives thereof (grey dashed arrow from F to B in Figure 3; [44]).

Eventually, MSA tools (Supplementary Note S1) use the base frequencies in each alignment column of the refined MSA to take error correction decisions; either by a simple majority vote or by one of the more sophisticated approaches discussed in the section ‘Denoising with statistical error models’ and the sections right after it.

### k-mer frequencies and spectrum

In parallel, an approach based on *k*-mer frequencies for determining base frequencies in positional pileups was developed and has dominated the field of error correction. The idea was originally introduced in the EULER assembler for Sanger reads in 2001 [45] and, initially, it mostly co-evolved with the assembler versions of EULER.

**Figure 4.**
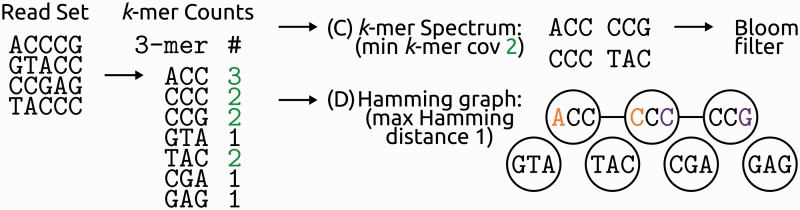
Deriving a *k*-mer Spectrum or a Hamming graph from *k*-mer counts. Some error correction tools work directly with the *k*-mer frequencies as counted from the read set. Others set a minimum *k*-mer coverage (2 in this example, green) to consider a *k*-mer as correct (trusted *k*-mers, green counts) and then derive a (C) *k*-mer Spectrum of all trusted *k*-mers. In this simplified example, this step classifies *k*-mers from the end of the queried sequence as untrusted (the reference in Figure 3A could be considered the sequence queried by the four reads). By using a Bloom filter, space usage of the *k*-mer spectrum can be reduced. Another concept used in *k*-mer approaches is the Hamming graph, where nodes are *k*-mers from the read set and nodes are connected if the Hamming distance (i.e. the number of base substitutions between them; see also the section ‘Substitutions only versus substitutions plus indels: Hamming versus Levenshtein distance’) is below a given threshold. In this simplified example, *k*-mers are too short and the Hamming graph therefore connects three correct *k*-mers. In a real setting, the *k*-mer length must be chosen with care (section ‘Optimal k-mer length’) and most connected components of the Hamming graph should contain only a single correct *k*-mer plus *k*-mers generated from the same sequence with errors. A colour version of this figure is available at BIB online: http://bib.oxfordjournals.org.

As for the seed subsequences of the pairwise read alignments in MSA approaches, each *k*-mer that exists in the set of all available reads is recorded. But instead of storing the information in which reads a *k*-mer occurs, only the total number of occurrences of the *k*-mer in the read set is counted and saved. This gives one ‘coverage’ (occurrences) value for each *k*-mer (e.g. *k*-mer counts table in Figures 3 and 4). Using the assumption that errors are rare and random, a threshold is then chosen to distinguish between low-frequency *k*-mers that are considered ‘erroneous’ (or ‘weak’ or ‘untrusted’) and high-frequency *k*-mers (‘solid’, ‘strong’ or ‘trusted’; green counts in Figure 4)—this approximates what is called the actual ‘*k*-mer spectrum’ of the sequenced sample (Figure 4C). The original *k*-mer spectrum correction approach was to inspect one full read at a time and to consider it erroneous, if it contains at least one untrusted *k*-mer—in other words, if the *k*-mer spectrum of the read contains *k*-mers not in the trusted spectrum of the full sequenced sample. Such a read is either corrected by the minimum number of base edits necessary to turn all of its untrusted *k*-mers into trusted ones (see also the section ‘Substitutions only versus substitutions plus indels: Hamming versus Levenshtein distance’), or discarded if that is not possible without making more than a pre-defined maximum number of changes to the read in question. Many different error correction approaches using the *k*-mer spectrum (Supplementary Note S2) or just the *k*-mer frequencies have been developed. We also discuss these further in relation to the choices for the frequency thresholds and the *k*-mer length (sections ‘Global frequency thresholds for trusting *k*-mers’ and ‘Optimal *k*-mer length’).

### Suffix tries and arrays, the Burrows Wheeler transform and the Full-text index in Minute space

**Figure 5.**
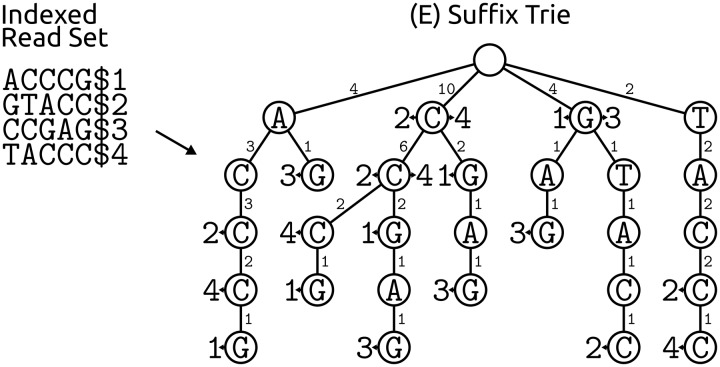
A suffix trie is a tree of all suffixes from the indexed example read set. Every existing suffix can be spelled out by a path from the root node to one of the read indices, indicated by arrowheads and read numbers at corresponding nodes. Numbers at trie edges correspond to the number of suffixes passing through them, i.e. edge weights give the coverage of a sequence from the root down to the following node. For example, the 2-mer ‘CC’ occurs six times in the four example reads.

**Figure 6.**
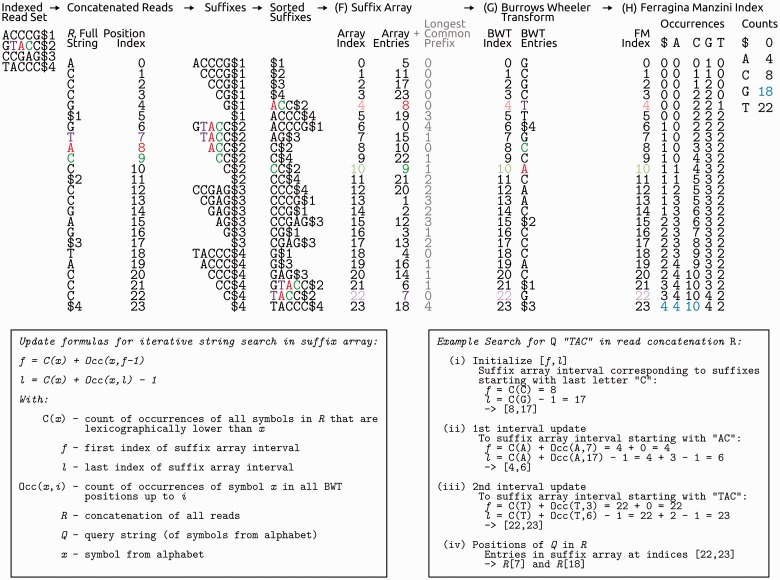
Steps for deriving first a suffix array and then the BWT and the FM index from the running example read set. Also given is an example for a string search using BWT and FM index, with the colours purple, red and green tracing corresponding indices and nucleotides. For suffix array construction, a unique termination symbol ($*x*) is appended to each read and reads are concatenated to a string *R* in lexicographical order of their termination symbol ($1<$2 < $3 < $4). All possible suffixes are formed and sorted unambiguously, as termination symbols have an order, as do the other symbols $ < A < C < G < T. A suffix array entry at suffix array index *i* then corresponds to the position in string *R* at which the *i*-th (lexicographically) lowest suffix starts. The LCP of a suffix array entry and the preceding entry is then recorded and suffix array plus LCP already form an efficient data structure for determining string occurrence frequencies. The BWT enables further compression of the data. Here, an entry at BWT index *i* corresponds to the symbol before the *i*-th lowest suffix in *R.* Together with the FM index, it allows for linear time string searches (and thus also determination of the coverage of a string) in the whole read set. The FM index gives the number of occurrences of each of the symbols up to any index of the BWT and for each symbol counts all occurrences of all lexicographically lower symbols (e.g. 4 + 4 + 10 = 18 for symbol ‘G’, all counts in blue). A colour version of this figure is available at BIB online: http://bib.oxfordjournals.org.

A third popular approach for determining base frequencies per sequence position uses data structures based on read suffixes: suffix tries, suffix arrays and their derivatives (Figures 3E, 3F, 5 and 6). A suffix trie, used in (Hybrid) SHREC [46, 47], is a tree of all possible suffixes of all reads, where each edge is weighted by the number of reads that support it (Figure 5). While a suffix trie requires considerable memory (determined by the length of the sequenced genetic material, the length of the reads and the amount of errors), it allows for very quick string searches of the whole data set. Also, the weights enumerate the number of read suffixes sharing the same prefix down to that level in the tree and if we regard the inspected level of the tree as the length *k* of a *k*-mer, the trie directly provides the frequencies of all possible *k*-mer lengths from 1 to the maximal read length. However, not all levels of the trie are useful for error correction, and only intermediate levels of the trie are inspected to check for imbalances in the weights of edges at each node (for a discussion of the sweet spot in *k*-mer length, see the section ‘Optimal *k*-mer length’ and Supplementary Note S3).

A suffix array [48], first used by HiTEC [49], is a sorted array of all possible read suffixes and is usually combined with an auxiliary index: the longest common prefix (LCP, Figure 6F). For a given position in a suffix array, the LCP gives the length of the longest common prefix that the respective suffix shares with the suffix preceding it in the array. With these two data structures, which require considerably less space than a suffix trie, *k*-mer frequencies can be queried for *k*-mers (called ‘witnesses’ in HiTEC) of varying length as easily as in a suffix trie. HiTEC exploits this property to iterate over various *k*-mer lengths that are chosen to optimize correction (section ‘Optimal *k*-mer length’ and Supplementary Note S3). An optimization idea—first introduced for suffix trees [50]—was recently implemented for suffix arrays in PSAEC [51] and Fiona [52]: they construct only a partial suffix array up to a specified order *h*, in a process they parallelize. This effectively means that suffixes are only sorted according to their first *h* bases, which still allows *k*-mer frequencies to be determined for all *k*-mer lengths up to *h.*

In the SGA assembler [44], a different optimized data structure, the Full-text index in Minute space (FM index; invented by Ferragina and Manzini; Figure 6H; [53]), was employed for error correction—a data structure originally introduced for string-graph construction in *de novo* assembly. The FM index uses the Burrows Wheeler transform (BWT, a lossless compression of a suffix array; Figure 6G; [54]) in conjunction with two auxiliary arrays: The cumulative occurrence of every symbol in the BWT is counted in the order of the BWT index (the Occurrences table in Figure 6H), and the total count of all bases that are lexicographically lower than a certain base in the full BWT is recorded (the five Counts in Figure 6H, one count for each symbol, cumulative in the lexicographical order). In the assembler fermi [55], the FM index was further optimized to incorporate both of the reverse complement strands of DNA in one index, the FMD index, which allows for bidirectional match extension in a pattern search.

## Error detection and correction: telling true polymorphisms from errors

### Substitutions only versus substitutions plus indels: Hamming versus Levenshtein distance

Instead of the Levenshtein edit distance, which allows measurement of single nucleotide insertions, deletions and substitutions [56], most error correction tools use the Hamming distance [57], which accounts for substitutions only. This is usually justified by two main arguments: firstly, the number of substitution errors is an order of magnitude higher than indel errors in the predominant Illumina data and, secondly, the computational complexity of the approaches using only the Hamming distance is lower, which is especially important for error correction procedures in high-throughput data and computation intensive tasks like *de novo* assembly. As a result, most tools only correct substitution errors (Table 3), and some even make the Hamming distance their central concept: the *k*-mer frequency tools Reptile [58], Hammer [59] and BayesHammer [60] create a Hamming graph in which *k*-mers as nodes are connected by edges, if the Hamming distance between them is below a certain very low threshold (Figure 4D). Such connections can be easily found by proximity searches in different copies of the list of all *k*-mer that are each sorted systematically, ignoring a certain amount of positions, where the number of positions disregarded in the sorting of each copy determines the Hamming distance allowed. A similar approach in the MSA tools Coral [61] and ECHO [62] is the use of adjacency lists. And finally, FreClu [21] and an unnamed tool by Aita *et al.* [63] cluster reads by their Hamming distance and then use different error correction strategies on the resulting neighbourhoods (i.e. the clusters).

On the other hand, only few tools (Table 3) explicitly implement indel correction: in MSA tools, this can be accomplished by creating or optimizing the MSA with a pairwise alignment algorithm that allows for gaps (e.g. [37]). This is the case in most MSA error correction tools and only some more recent tools trade the indel correction capability for computational speed: SGA [44] uses an optimized data structure (the BWT representation of the suffix array, Figure 6) and creates MSAs from it with an algorithm that does not natively support indel detection. ECHO [62] has not implemented indel correction to save computational resources.

A recent approach, implemented in Fiona [52], is to optimize correction possibilities over the three different single nucleotide error types by finding optimal alignment extensions from suffix array seeds between an erroneous and all the corresponding correct reads. This implements the Levenshtein distance at the error position and even allows for further insertions or deletions in the alignment extension that are then penalized in the optimization.

In general, the decision whether to implement an error correction of indel errors is becoming increasingly important, as the emerging platforms by Ion Torrent, PacBio and Oxford Nanopore produce a substantial amount of indel errors (Table 1 and Figure 2). This is further exemplified by recent *k*-mer tools such as Blue [64], which explicitly consider indels, despite the increased computational complexity.

### Global frequency thresholds for trusting k-mers

Many *k*-mer frequency tools create a *k*-mer spectrum to correct untrusted *k*-mers to the closest trusted one, usually by minimizing the Hamming distance between them (i.e. substitutions only, section ‘Substitutions only versus substitutions plus indels: Hamming versus Levenshtein distance’). To this end, they use the assumptions that errors are rare, that different errors are equally likely and that coverage is uniform, and rely on a frequency threshold to decide which *k*-mers to trust (e.g. two in Figure 4). While this threshold was originally chosen manually from experience—e.g. in EULER [45, 65] and SOAPdenovo [66, 67]—more objective criteria to derive the threshold from the actual data were soon developed (Table 3, Supplementary Table S2).

**Figure 7.**
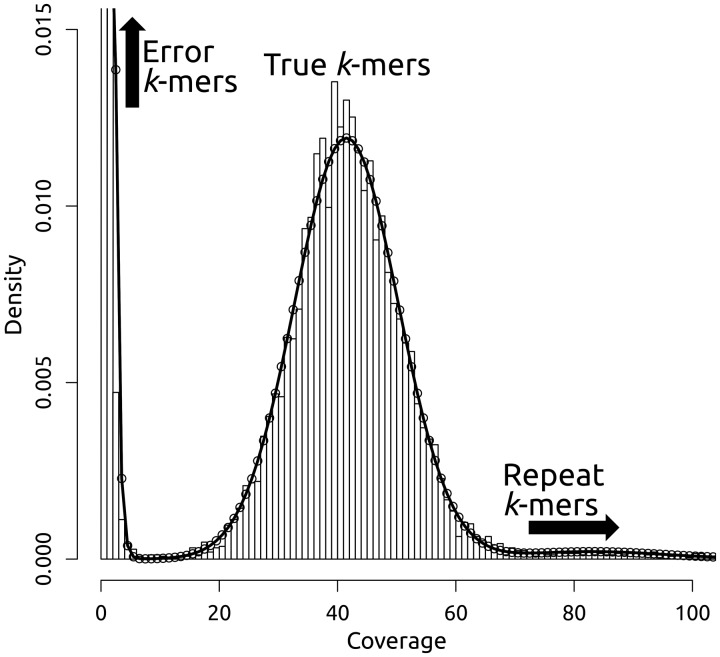
*k*-mer coverage histogram with a model fit. The histogram in this plot from the Quake paper [70] gives a nice example of an empirical *k*-mer coverage distribution. The density tells us which proportion of all existing *k*-mers in the data set has a particular coverage. The solid line gives the Quake model fit. The first peak of the distribution is formed by very low coverage error *k*-mers and is usually modelled by a Poisson or a Gamma distribution. The second peak results from the majority of correct *k*-mers and is usually modelled by a Poisson or a Gaussian distribution. Between these two peaks, a clear local minimum can provide a *k*-mer trust coverage cut-off. The heavy tail of higher multiplicity *k*-mers is the result of *k*-mers from sequence repeats. Quake draws the *k*-mers' sequence copy numbers from a Zeta distribution and then projects these *k*-mers into the coverage range of the correct *k*-mers. Adapted by font change and label addition from [70], according to the Creative Commons Attribution license CC-BY 2.0 (http://creativecommons.org/licenses/by/2.0/).

In EULER-SR, the threshold was chosen manually, but an explicit reasoning for the choice was provided [68]: given the number of reads (*N*), their average length (*L*), an approximate genome size (*G*) and the *k*-mer length (named *l* in the original publications), an average *k*-mer coverage can be computed as: *a* = *N* * (*L* − *l*) / *G.* The coverage of correct *k*-mers is then assumed to follow a Poisson distribution around that average *a,* and the coverage threshold is chosen such that only very few correct *k*-mers (e.g. less than 100 *k*-mers in a full data set) will theoretically fall below that threshold. Along similar lines, ALLPATHS [69] assumed two distinct distributions underlying the empirical distribution: one for erroneous *k*-mers (with a very low frequency, Figure 7) and one for correct *k*-mers. The peaks of these two distributions were consistently found to be separated by the first local minimum of the empirical overall *k*-mer distribution and this first local minimum was therefore taken as the threshold (Figure 7). Most subsequently developed tools adopted similar strategies, with some refining it further (for the nuances of the global *k*-mer frequency threshold determination across tools, see Supplementary Note S4). Most notable in this respect is Quake [70]: here, *k*-mer frequencies are weighted by quality values (producing ‘*q*-mers’) to more clearly separate the empirical distribution maxima. In addition, a third distribution accommodates for the heavy tail of high multiplicity *k*-mers from repeats in the queried sequence (Figure 7). Quake projects these repeat *k*-mers into the distribution for correct *q*-mers and then uses a maximum likelihood fit of its full mixture model to determine a *q*-mer frequency threshold (Supplementary Note S4).

A major caveat of the approaches relying on separable *k*-mer distributions is that they are only applicable when coverage is uniformly distributed over the queried sequence. Therefore, the here mentioned error correction tools (and any tool with a global *k*-mer trust threshold; Table 3) are not applicable to data sets with inherently variant coverages, such as in metagenomics and transcriptomics, or with strongly biased coverages (e.g. owing to the GC bias of the platforms used or owing to bias-prone whole genome amplification of samples, as in single cell sequencing). To correct such data sets, the software presented in the section ‘Removing the uniformity of coverage assumption’ can be considered.

### Optimal k-mer length

**Figure 8.**
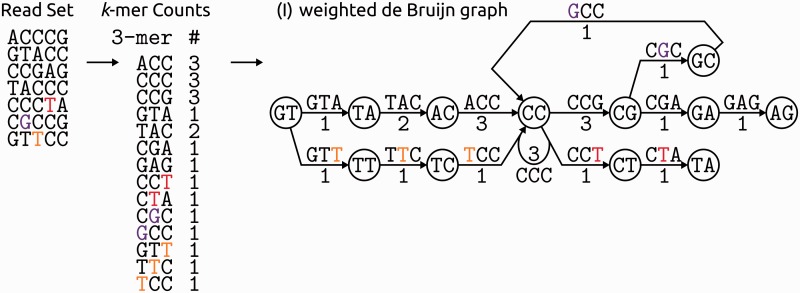
Example of a weighted de Bruijn graph from the example read set. The read set is augmented to include reads that show variation compared to the example read set in the earlier figures: while the Ts (red and orange) could be substitution errors and the G (purple) could be an insertion error, all three could also be reads covering alternative alleles of the same sequence locus or slightly different repeats at other sequence loci. The new reads create graph structures that are commonly removed in graph pruning (and thus correction) steps: (i) the orange T creates a bulge, a cycle in the graph that can not be traversed by a single path, as edges of the cycle go in opposing directions (e.g. edges ACC and TCC both ending at node CC); (ii) the red T creates a tip, a short dead end of the graph; (iii) the purple G creates a whirl, a cycle in the graph with a possible path going around the whole cycle (i.e. all edges go in the same direction). A repeat graph would have small (and assumingly erroneous) whirls, bulges and tips removed and repeats collapsed, but care must be taken to not remove genuine variation. A colour version of this figure is available at BIB online: http://bib.oxfordjournals.org.

Software that uses *k*-mer frequencies has to choose a specific *k*-mer length (e.g. three in Figures 3, 4 and 8). The trade-off inherent to this *k*-mer length choice—an equally important question for de Bruijn graph (Figure 8) assemblers—was first discussed in detail in the paper describing Quake [70]: ‘Smaller values of *k* provide greater discriminative power for identifying the location of errors in the reads and allow the algorithm to run faster. However, *k* cannot be so small that there is a high probability that one *k*-mer in the genome would be similar to another *k*-mer in the genome after a single nucleotide substitution because these occurrences confound error detection.’, i.e. each *k*-mer has to be long enough to be unique in the queried sequence (assuming that every *k*-mer is equally likely to occur at any position throughout that sequence), but if a tool can only detect one error per *k*-mer—the default setting for most tools, as they use a Hamming distance of one between *k*-mers—a longer *k*-mer means a lower error resolution. To determine the length, usually the user is asked to provide a *k*-mer length (sometimes with some guidance as by the ‘sga stats’ functionality of SGA; [44]) or the software provides a default value that was found to work well in practice (Table 3). In contrast, the authors of Quake analysed their above statement of the trade-off more systematically and recommend the following for setting the *k*-mer length: ‘[T]he probability that a randomly selected *k*-mer *k* from the space of (4^*k*)/2 […] possible *k*-mers occurs in a random sequence of nucleotides the size of the sequenced genome *G*, is ∼0.01’. Based on this requirement, they give an approximation of *k* ≈ log_4(200*G). This translated into recommended *k*-mer lengths of 15 for the ∼5 Mb *E. coli* genome and 19 for the ∼3 Gb human genome. However, longer *k*-mers are useful for a better resolution of repeats in error correction and are now becoming manageable: longer and more accurate reads on average give more correct *k*-mers per individual read; deeper sequencing gives high enough *k*-mer coverage for correction decisions on longer *k*-mers (otherwise a problem, especially for assembly contiguity [71]); and newer implementations and hardware enable the handling of the resulting *k*-mer spectra.

Further examples of an explicitly motivated *k*-mer length choice come from the realm of suffix tries and arrays (Figures 5 and 6), where the data structures natively allow for the inspection of several different values of *k.* (Hybrid) SHREC [46, 47] only inspects node weights at intermediate levels of the tree, corresponding to intermediate *k*-mer lengths, where tree height is determined by the read length. HiTEC [49] determines the values of *k* that minimize the probability of false-negative corrections and those that minimize the probability of false-positive corrections. It then uses multiple *k*-mer lengths around those two optima. Fiona [52] also adopts this approach and further adapts it to account for varying read lengths. Altogether, these approaches automatically determine the *k*-mer lengths they use and are less prone to *k*-mer length effects as they use multiple *k*-mer lengths within a correction run (details in Supplementary Note S3).

Finally, a recent tool originally aimed at *k*-mer length determination for assembly with de Bruijn graphs, called KmerGenie [72], has the potential to also automate the determination of optimal *k*-mer lengths for *k*-mer-based error correction. It uses an extension of Quake's *k*-mer distribution model that it fits to the empirical *k*-mer count distribution for different values of *k* separately. This model fit estimates the number of distinct correct *k*-mers for each *k* (i.e. *k*-mers of that length that would be in a correct reference) and the *k* with the largest number of correct *k*-mers is considered optimal. To make the *k*-mer counting for the empirical *k*-mer distributions computationally tractable for multiple values of *k* (regarding both runtime and memory consumption), KmerGenie subsamples the *k*-mers by a factor *ε* (the authors used *ε* = 1000).

### Removing the uniformity of coverage assumption

As discussed above, the *k*-mer approaches mostly rely on a global threshold for the decision if a *k*-mer count is to be trusted and such a threshold makes a very strong assumption, namely, that coverage is uniform across the whole queried sequence. Thus, this method will mis-classify *k*-mers from low coverage regions that do exist in the query as untrusted, no matter how sophisticated the threshold determination is, and will equally fail on data sets with inherently variant coverages. The automatic determination of a global threshold from the empirical *k*-mer frequency distribution is even discouraged for uneven coverage for Quake [70], and will usually fail: no discernible histogram peaks of correct and erroneous *k*-mers (Figure 7) can be found in data with non-uniform coverage.

For this reason, some newer *k*-mer-based tools avoid the use of such a global threshold, and instead use one of the following strategies:

- Reptile [58], Hammer [59], BayesHammer [60] and a tool by Sleep *et al.* [73] implicitly group reads by their Hamming distance, by creating a Hamming graph of *k*-mers (for Hamming distance and Hamming graph, see also the section ‘Substitutions only versus substitutions plus indels: Hamming versus Levenshtein distance’ and Figure 4D). In the Hamming graph, they examine connected components of very similar *k*-mers, calling them a *k*-mer neighbourhood. Based on the relative frequencies of *k*-mers within such a Hamming neighbourhood, Hammer chooses a consensus *k*-mer for each connected component, thus taking one local decision per Hamming neighbourhood and outputs corrected *k*-mers. BayesHammer subdivides Hamming neighbourhoods further by *k*-means clustering, with the subclusters' probabilities approximated by the contained reads' sequencing quality scores. This subclustering distinguishes very similar *k*-mers that initially share a neighbourhood but originate from distinct sequence locations (e.g. repeats), but should not create new subclusters from erroneous *k*-mers. It then creates a *k*-mer spectrum from high-quality clusters, expands it based on high-quality reads and does a read-based correction. Reptile also focuses on the read as a unit in its correction approach. It inspects several *k*-mers of each read in an order specified by what they name a ‘tiling’, searches for higher-frequency *k*-mers within the respective Hamming neighbourhoods and finally corrects a read to an alternative tiling (a set of alternative *k*-mers) if it has a substantially higher coverage than the respective set of *k*-mers of the original read. Thus, this correction decision integrates more contextual information than Hammer and does, at least locally, rely on a more uniform coverage.
- Blue [64] uses a *k*-mer spectrum (which initially uses a very low global exclusion threshold), but creates its *k*-mer trust threshold for correction for each read separately. In this way, it adapts it to the local coverage and allows for *k*-mer coverage variations between reads.
- Trowel [74] does not use a coverage threshold for its *k*-mer spectrum at all. Instead, it initially includes only *k*-mers whose bases all have quality values within the top 8% of the data set in at least one read and then iteratively expands the spectrum by ‘boosting’ the quality values of corrected bases to the maximum quality value known for the correcting *k*-mer. In this way, no coverage assumptions are made at all.
- EDAR [75], like BayesHammer, Reptile and Blue, also focuses on one read at a time in its correction approach. It classifies regions of each read, thereby circumventing a global threshold. Using the variable bandwidth mean-shift method, it identifies contiguous stretches along a read with consistent *k*-mer coverage and identifies breakpoints where the coverage rapidly changes to a different consistent level (with the unique feature of a GC-bias correction of the *k*-mer frequencies). Then, it classifies read regions—instead of *k*-mers or full reads—as untrusted (very low coverage) or as trusted and further subdivides the trusted regions into unique and repeat regions (very high coverage). This is done by inherently local clustering decisions (Figure 1 of [75]).
- Not looking at full *k*-mers at a time, but instead only at one base column of a pileup, also avoids a global threshold altogether: decisions can be taken on relative base frequencies at individual positions with no assumptions about global coverage uniformity. QuorUM [76] does just this: it uses a refined voting scheme relying on the base frequencies at each position. ALLPATHS-LG [77] and fermi [55] further weight these frequencies with the associated quality scores from the original reads, adapting Quake's ‘*q*-mer’ idea to the individual pileup position (compare with the section ‘Global frequency thresholds for trusting *k*-mers’).

What requires a special focus on single positions instead of *k*-mers in the *k*-mer tools comes more natural to the MSA approaches: a global threshold can easily be avoided, as a decision can be taken directly on relative frequencies at each base column of the alignment. However, SGA's [44] MSA module nevertheless uses a global threshold for the sake of simplicity, but all the other MSA tools conduct some sort of column-based majority voting or statistical testing, effectively taking genuinely local decisions.

Finally, the two hidden Markov model (HMM)-based error correction approaches, SEECER [78] and PREMIER [79], also take inherently local decisions with their emission probabilities derived from MSA alignment positions or *k*-mers, respectively.

Altogether, the software described here is especially interesting for sequencing data sets with non-uniform coverage, such as in transcriptomics, metagenomics and single cell genomics. But however important it may be to avoid the assumption of uniform coverage, taking local decisions (often at one query position at a time) will usually ignore contextual information. We have already glimpsed at the use of such context information over the range of a whole read in BayesHammer, Reptile and EDAR in this section. But a more exhaustive account of how tools use a longer context range around each inspected position for their correction decisions is given in the section ‘Repeat and haplotype models’.

### Denoising with statistical error models

Whereas most tools have no error model at all, others accommodate for some of the error biases we have reviewed to more precisely distinguish between sequencing errors and genuine sequence variation at low frequencies. The simplest error models use a global (i.e. uniform) error probability that is empirically known for the respective sequencing platform or take the assigned PHRED quality score as a substitute for a local error probability (e.g. [60, 63, 74]), as they have been proven to generally correlate with sequencing errors for most platforms. However, as we have reviewed, error frequencies are known to be biased by platform specifics (different base confusion probabilities and signal decay throughout a read), local sequence content (GC content or sequence motifs, such as homopolymer stretches and inverted repeats) and preparation steps that precede the sequencing protocol (e.g. pre-amplification).

Therefore, an approach used by several tools (Supplementary Note S5) is to employ an empirically determined base confusion matrix that gives the probability of every possible base substitution separately instead of assuming a uniform substitution probability (i.e. a matrix with 4 by 4 substitution probabilities). Here, a general known matrix for the respective platform can be used, but most tools learn the matrix from the data and incorporate further features (Supplementary Note S5). An analogous concept for 454 pyrosequencing, where homopolymer length miscalls are the most prominent error type, is to use a distribution of light intensities per homopolymer length that has been determined empirically from sequencing of known sequences, e.g. by Balzer *et al.* [18].

Several tools also use other specific approaches to estimate errors: AutoEdit [80] combines information from the alignment of a read with three measures of the peak resolution of its original capillary sequencing flowgram, to determine the probability if a base call was correct. RECOUNT [81] calculates error probabilities for each position in each read by taking the quality value average of that positions alignment column. And pacbio_qc [28], aimed at CCS with the PacBio RS platform, aggregates the number of reading passes over a read (a special feature of CCS) and the mean quality value into an error probability.

Finally, three tools use more refined error models that are worth a more detailed look:

- The authors of SysCall [82] first analysed error occurrences and identified a new type of systematic error when using Illumina platforms. These errors show: (a) a drop in coverage compared with surrounding bases, (b) a very specific base confusion matrix at the error site and a bias in the two bases directly upstream and (c) a strong strand bias. Based on these characteristics, they trained a logistic regression classifier to distinguish genuine heterozygosity from such errors at potential heterozygosity sites, using reads from known sequences as training data.
- PREMIER [79] fits a HMM of *k*-mer transition probabilities across all reads in the data. This probability, for a certain position within a read, to transition to the next position, given a certain emission *k*-mer, is derived from four values: (a) the position within the read, (b) the probability of a substitution error at this position given the quality value at that position, (c) a general base confusion matrix and (d) the count of the possible emission *k*-mer in the data. However, the authors do not provide any benchmarking regarding runtimes, suggesting that the approach might not scale to larger sequencing data sets.
- Fiona [52] employs a hierarchical statistical model of the sequencing process for its decision whether a position is erroneous. Using the assumptions of a uniform coverage and a uniform error probability, it determines the log odds ratio of an error given the observed *k*-mer coverage of a specific possible error *k*-mer and the general log odds ratio of an error in any *k*-mer. If the log odds ratio of an error in the specific *k*-mer is higher than the log odds ratio of an error in a general *k*-mer, the *k*-mer is considered erroneous (more details of the model in Supplementary Note S6). As the authors note, this threshold can easily be varied, if more appropriate thresholds are found or a different sensitivity of error correction is required. But not only this aspect of the model can easily be adjusted, making the model an attractive starting point for the development of more flexible error correction tools in the future.

In summary, many error correction tools do not model errors statistically—usually for the sake of simplicity. At the same time, the few tools presented here that do incorporate some sort of error model show considerable diversity in their approaches, with some form of standard or learned base confusion matrix at the heart of most models. Especially in platforms like Illumina's, where errors are well characterized, read error correction could benefit from such more specific approaches.

### Repeat and haplotype models

The central issue in sequencing error correction is to distinguish errors from genuine variation, no matter if such variation is owing to repeats within a genome, or different alleles or haplotypes within a population or a single cell. Most tools are based on the assumptions that errors are rare and random and that coverage is uniform, while others ignore reads or *k*-mers from repetitive regions in their correction procedures, e.g. Fiona [52]. We describe four approaches that model the expected footprint of genuine variation to make decisions clearer: (i) ECHO models a diploid genome; (ii) EULER-USR provides a repeat model by using the repeat graph; (iii) MisEd, SGA, Acacia and SEECER all determine whether mismatches between a read and the consensus are linked within reads to disentangle inexact repeats; (iv) ShoRAH assigns reads to an arbitrary number of haplotypes using a Gibbs sampler.

**Figure 9.**
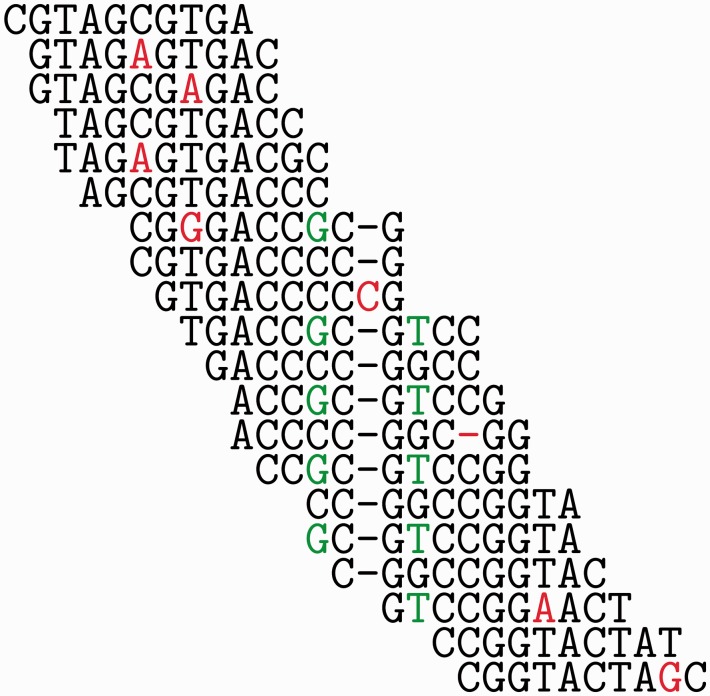
Example of a MSA of a read set, demonstrating consistent mismatches (green nucleotides) in comparison to isolated or low-frequency mismatches and indels (red nucleotides and dashes). The consistency of mismatches can be tested through their linkage within reads (four in this example) and are called DNPs in the first tool that used such information, MisEd [86, 87]. However, genuine polymorphisms can only be told from sequencing errors if multiple of the linked variant sites are within the range covered by the average read (pair). A colour version of this figure is available at BIB online: http://bib.oxfordjournals.org.

- ECHO [62] maximizes an a posteriori estimate of each possible genotype at a particular read position, given a prior probability that this site is heterozygous (versus being homozygous) and given the base frequencies from a MSA containing the read position. For a haploid genome, this probability can simply be set to 0. Therefore, this model effectively allows for one or two haplotypes at each inspected site.
- EULER-USR [83] uses its assembly data structure, the repeat graph, to make its error correction repeat-aware. First, the read prefixes are error corrected using a simple *k*-mer spectrum approach, as they are deemed the most accurate part of reads (error rates increase with read length in most platforms, see the section ‘Sequencing Platforms and their Errors’). Then, an A-Bruijn graph is constructed from the corrected read prefixes: this is a generalized version of the de Bruijn graph (Figure 8), where all edges connecting the same vertices are summarized into one edge with a weight reflecting the number of original edges covering it [84]. Inconsistent graph structures are removed (i.e. bulges, tips and whirls, Figure 8), which effectively amounts to the elimination of sequencing errors from the graph and the only remaining tangles in the simplified graph should be genuine repeats (or haplotypes), thereby giving the repeat graph [84]. The less accurate read ends can then be corrected by mapping the whole read into this read prefix repeat graph and correcting it to the consensus. A similar approach is taken by the recent PacBio hybrid error corrector LoRDEC [85]: here the long error-prone PacBio reads are corrected by threading them through a de Bruijn graph constructed from more accurate short reads from a different platform (see also the section ‘Platform specific error correction’). This graph-based error correction approach and its graph abstraction—designed with *de novo* assembly in mind and not population resequencing—models repeats (or heterozygosity) within a genome implicitly but will most likely err on the side of removing low-coverage polymorphisms.
- MisEd [86] was the first tool to capitalize on the fact that, given long enough reads, genuine discrepancies stemming from (inexact) repeats will consistently be linked across several reads, while errors (assumed to occur rarely and at random) should be isolated on single or at least fewer reads (Figure 9). To this end, they used their previously developed concept of defined nucleotide positions (DNPs; [87]): Columns with a deviation from the (MSA) consensus are considered candidates, and pairs of candidates are tested for significant co-occurrence within reads, i.e. they approximate the expected amount of coincidences between two sites, given the number of reads covering both sites, and model the respective quality values at the sites as Poisson distributed. From this, they compute the probability of observing at least the seen amount of reads that share both deviations from the consensus and regard the two sites as DNPs if this probability exceeds a pre-defined threshold. MisEd does a DNP analysis of regions with a higher than expected amount of mismatches relative to the MSA consensus and the subsequent error correction, done by consensus calling over every column of the MSA, skips the identified DNPs as genuine repeats. MisEd thus uses an explicit repeat model that protects all the repeat (or haplotype) variation it can identify from false-positive error corrections. The MSA module of the SGA assembler [44] uses a similar, but simpler, check before correction: it identifies conflicting columns in the MSA as columns where at least two different bases surpass a given minimum coverage threshold. It then excludes reads from the original alignment before performing the error correction, if they consistently differ from the consensus at all conflict columns they cover and thus effectively incorporates a simpler repeat model than MisEd. Acacia [88], a tool developed for 454 pyrosequencing data (section ‘Platform specific error correction’), also uses linkage between mismatches as in SGA. However, it clusters and aligns run-length encoded reads (i.e. collapsing all their homopolymer stretches) and then identifies alignment columns with significant differences in homopolymer length between reads (section ‘Platform specific error correction’). Reads that share several of such significant homopolymer length differences across an alignment are separated into a new cluster that is fed back into the original process. SEECER [78], a tool mainly aimed at RNAseq data, works along similar lines: it refines MSA contigs by separating different inexact repeats (or haplotypes) within the initial contig. Columns with mismatches to the MSA consensus are identified and clustered by the set of reads sharing the respective mismatch (spectral clustering and spectral relaxation of *k*-means). With this approach, consistent and homogeneous subsets of reads can be separated for further analyses and the clustering thus implies a model of repeats and possibly haplotypes (more SEECER details in Supplementary Note S7).
- ShoRAH [41, 42] aims at heterogeneous samples containing many haplotypes of the same species, like viral communities of quasispecies. It inspects overlapping windows of defined length (chosen so that each position is covered by three distinct windows) in a mapping-derived MSA and then reconstructs their (local) haplotypes. To this end, the reads in that window are clustered into haplotypes using a Gibbs sampler to iteratively draw from the posterior distribution of a Dirichlet process mixture (DPM). The DPM assumes a prior probability of assigning a read either to an existing haplotype or to a newly instantiated one. Around this prior, it builds a haplotype model, effectively generating a multivariate posterior distribution over: (a) the read assignments to haplotypes, (b) the sequence of the haplotypes, (c) the per-position probability of a correct base call (i.e. one minus the probability that a sequencing error occurs at any random position) and (d) the per-position probability of a haplotype being identical to the reference at that position (i.e. one minus the probability that a mutation occurred between reference and haplotype at any position). This iterative sampling eventually leads to a stable population of clusters that represent haplotypes, and correction is then done by a majority vote over the haplotype reconstructions from the three different window sets that overlap each MSA position. Altogether, the clustering in ShoRAH explicitly models an arbitrary number of haplotypes.

In summary, four approaches to model genuine variation in similar sequences exist in error correction tools: (i) The model of a diploid genome in ECHO. However, this is neither applicable to (mixtures of) populations nor if multiple inexact repeats appear within a genome. (ii) The model of the repeat graph in EULER-USR (and LoRDEC). This can accommodate an arbitrary number of inexact repeats and/or haplotypes, but is aimed at *de novo* assembly and will thus probably underestimate diversity, especially in the face of non-uniform coverage of different haplotypes or repeat copies. (iii) The linkage of mismatches (to a consensus) within a read is examined by MisEd, SGA, Acacia and SEECER, with an approach that implicitly models inexact repeats. This will also work for haplotypes when several of the respective polymorphisms distinguishing haplotypes lie (well) within the distance covered by a read (or a read pair in paired-end sequencing), but will fail to protect isolated polymorphisms (e.g. in similar long repeats or closely related strains in a population) from false-positive correction. (iv) The most flexible haplotype model that can accommodate an arbitrary number of haplotypes is implemented in ShoRAH. It could thus be well suited not only for error correction in viral quasispecies community sequencing but also in metagenomic sequencing data.

### Platform-specific error correction

#### 454 pyrosequencing

Some approaches consider the Levenshtein distance and thus include the correction of the prevalent indel errors of 454 pyrosequencing data. However, some tools have even been designed especially for this type of data, mostly for the smaller data sets from amplicon sequencing. The first tool in this field, PyroNoise [89], introduced the idea of flowgram clustering. Flowgrams record the light intensities from the consecutive flushes of the four different nucleotides across the sequencing plate, and the total amount of consecutive integrations of the respective nucleotide (i.e. the homopolymer length) can be determined by rounding the light intensities to integers. However, this process is error prone and Quince *et al.* [89] thus went back to the flowgrams for their error correction. They use a mixture model of light intensities (of a single flush) for different homopolymer lengths with probability distributions learned from sequencing of known sequences. On this, they apply an expectation-maximization algorithm to maximize the likelihood that the observed set of flowgrams (and their frequencies) was generated from an assumed set of true sequences (with one cluster per assumed true sequence). Eventually, the most abundant read in a cluster is taken as the representative for all the contained reads. DeNoiser [90] increased the speed of this approach by using a heuristic for the construction of initial clusters: it first filters out reads that are prefixes of other reads and then uses the identified read prefix clusters, ordered by their abundance, as the starting point for a clustering. In contrast, AmpliconNoise [91] made the approach more accurate by also accounting for errors introduced in the PCR step of amplicon production, using a learned base confusion matrix (Supplementary Note S5).

Three other tools instead look at the reads after base calling: Acacia [88], HECTOR [92] and KEC [93]. The first two each use run-length encoding (RLE) on homopolymers. This idea of collapsing homopolymer runs into a single nucleotide to allow analysis of pyrosequencing data disregarding homopolymer length errors, was first introduced in a version of the Celera assembler [94]. Acacia [88] hashes RLE read prefixes (disregarding the run-length of individual homopolymers at this point), thus creating initial read clusters (reminiscent of DeNoiser's pre-clustering). The clusters are then refined by merging clusters whose consensuses have similar 6-mer spectra and by creating new clusters from read sets whose run-lengths at homopolymer sites differ significantly from the rest of the cluster. Eventually, all reads are corrected towards the consensus of their cluster. HECTOR [92] also uses the RLE, but instead of applying the clustering approach to the RLE sequences, it applies the *k*-mer spectrum approach (a ‘*k*-hopo spectrum’), including the automatic determination of a *k*-hopo coverage trust threshold from its empirical distribution (Supplementary Note S4). KEC [93] also uses a *k*-mer spectrum approach, but without a previous RLE encoding. Instead, it adopts the strategy of EDAR (compare the section ‘Removing the uniformity of coverage assumption’) that identifies erroneous read regions by clustering the *k*-mers of a read by their frequency with the variable bandwidth mean-shift method. But instead of just removing the identified erroneous regions, as EDAR does, KEC corrects them, taking homopolymer length errors into account.

#### PacBio

In the long reads from the PacBio platform, the very high overall error rate is the major challenge. Currently, the two major strategies to address this are to either use less error-prone short reads from another platform with enough coverage to correct the long PacBio reads (called the hybrid approach), or to exploit the fact that errors seem to be unbiased in this platform and can therefore all be corrected, given enough coverage.

The first approach was initially implemented in PBcR [95] as a stand-alone tool and as a pipeline stage in the assembler AHA [96], the latter developed in direct cooperation with the machine vendor. In both approaches, the more accurate short reads from another platform are mapped onto the long reads. AHA then simply corrects the long reads towards the consensus of the resulting short read mapping, whereas PBcR further optimizes the alignments, creating a short read MSA that is then used for the consensus calling. The tool LSC [97] improved the alignments in this approach, by using the idea of RLE—or homopolymer compression, as the authors call it—that had been used in 454 pyrosequencing error correction shortly before (compare with Acacia and HECTOR in the section right above). Mapping RLE short reads to RLE long reads disregards homopolymer length errors and thus improves the mapping sensitivity.

A very recent tool, proovread [98], instead improves the plain mapping approach: Firstly, it makes it more sensitive by using alignment penalty values adapted to the error profile (separate penalties for insertions, deletions, substitutions and gap elongation). Secondly, it makes it scalable by parallelizing the process. It looks at mappings of short reads onto individual long reads and uses an iterative mapping and correction procedure that gradually includes more reads and allows for mapping with more mismatches in each round. Thirdly, it recognizes and splits chimeric long reads.

ECTools [99], recently published via bioRxiv, pre-assembles the more accurate short reads into unitigs using the Celera Assembler. It then aligns the long reads against those unitigs, optimizes this alignment by solving the longest increasing subsequence problem and corrects towards the unitigs.

LoRDEC [85], another recent hybrid approach, also does a pre-assembly and draws upon an existing strategy: the idea of threading reads through a generalized and weighted de Bruijn graph, first introduced as a *k*-mer approach solely on short reads in EULER-USR [83]. Where EULER-USR used only the more accurate prefixes of short reads to build the graph (section ‘Repeat and haplotype models’), LoRDEC uses only the short reads from a different platform, as they are more accurate than the PacBio reads. And where EULER-USR then threads the full short reads through the graph to correct them (including their less accurate suffixes), LoRDEC threads the long PacBio reads.

For the nonhybrid PacBio error correction approach, PBcR was adapted to use the higher abundance shorter reads from a PacBio RS sequencing run to provide the coverage for correcting all reads, including the lower abundance longer reads from the same run [26]. This approach was also implemented in the vendors assembly pipeline, called HGAP at the time [100].

#### Oxford Nanopore

For this technology, only one tool has so far been described via a bioRxiv publication: Nanocorr [101]. With the error rates similar to earlier stages of PacBio development, it adopts a similar hybrid error correction approach. Here, BLAST is used to align short MiSeq Illumina reads to the long Oxford Nanopore reads. This alignment is then optimized similarly to the ECTools strategy (see right above; [99]) and finally, a consensus of the short read alignment is called as the corrected long read [101].

## Conclusions

The choice of an error correction tool depends strongly on the type of analysis one wants to perform and on the sequencing platform that generated the data, as assumptions of a particular approach might not hold for the data set at hand. In general, error correction has also been proven beneficial for single nucleotide variant (SNV) calling [70] and haplotype reconstruction [102], but the tools are mostly used before genome assembly to reduce the complexity of alignments and the size of intermediate data structures. Most end-users just rely on the built-in error correction approach of their assembler of choice, as there are almost no systematic and independent benchmarks of error correction tools that could justify a different choice (notable exceptions are [36, 103]). We therefore believe that context-specific benchmarks are needed for many different data types and applications, and propose the following six aspects for choosing tools to benchmark for a particular set-up (Tables 2 and 3 and Supplementary Table S2): First and most foremost, the tool needs to be freely available, as are 52 of the 60 here presented tools (Table 4 and Supplementary Table S2). Second, whether the tool needs to account for substitution errors only, or should also consider indel errors, depends on the sequencing platform that generated the data. For Illumina and Complete Genomics data, indel errors are not as relevant as they are for the 454, Ion Torrent and PacBio platforms. Third, if coverage is expected to vary over the queried sequence(s), as in transcriptomics, metagenomics, heterogeneous cell samples or pre-amplified libraries (e.g. amplicon or single cell sequencing), then most *k*-mer tools (and the associated automatic *k*-mer trust threshold) are ill-suited—except e.g. the newer tools using a Hamming graph and Hamming neighbourhoods. In contrast, most MSA tools will be applicable to such data (section ‘Removing the uniformity of coverage assumption’). Fourth, if your analysis is rather sensitive to single nucleotide errors (e.g. when calling SNVs), you might want to use a tool with a more sophisticated (and/or more specific) error model (e.g. tools in the section ‘Denoising with statistical error models’, Supplementary Notes S5 and S6), although this will usually come at a computational cost. Fifth, if your data set does not come from a homogeneous haploid sample, one of the tools with a repeat and/or haplotype model will probably improve error correction (e.g. tools in the section ‘Repeat and haplotype models’), but will also require more computational resources. Sixth, how important scalability is, mostly depends on the size of your data set and will also influence which type of tool, regarding the used data structure, can be used. However, for all major approaches—the MSA approach, the basic *k*-mer approach and the read suffix approach—the most recent tools use efficient implementations and should all scale to large data sets. Thus, in the end, the choice of a tool will rather depend on the requirements for error correction in each instance.

**Table 2.** Recommendations which tools to consider for benchmarking of which data and analysis types

Criterion	Property	Data sets	Tools to consider
Platform	454	Any (homopolymer errors!)	HECTOR, KEC, Acacia, AmpliconNoise, DeNoiser, PyroNoise
Platform	Oxford Nanopore	Any (very high error rate!)	Nanocorr
Platform	PacBio	Any (high error rate!, little bias)	proovread, LoRDEC, ECTools, pacbio_qc, PBcR, LSC, AHA
Data property	Non-uniform coverage	Metagenomics, transcriptomics, whole genome amplified	Trowel, Blue, BayesHammer, QuorUM, fermi, Hammer, ALLPATHS-LG, Reptile
Data property	mostly substitution errors	Complete Genomics, Illumina	BFC, Lighter, Trowel, BayesHammer, QuorUM, Musket, RACER, SGA, SOAPdenovo2, fermi, REDEEM, Hammer, SysCall, DecGPU, ECHO, HiTEC, ALLPATHS-LG, Reptile, CUDA-EC, SOAPdenovo, Quake, SHREC, FreClu, EULER-USR
Data property	indel errors prevalent	Any (esp. 454, Ion Torrent, PacBio)	Fiona, Blue, SEECER, Coral, ShoRAH, Hybrid-SHREC
Data property	many repeats or haplotypes	Metagenomics, complex genomes (e.g. eukaryotes)	SEECER, Acacia, SGA, SHoRAH, EULER-USR
Data property	two haplotypes	Diploid genome	ECHO
Analysis type	sensitive to single nucleotide errors	e.g. for SNV analysis	Fiona, REDEEM, ECHO, SysCall, Quake, FreClu

Tools are given in chronological order of publication, newest tools first. Recommendations are based on this literature review of the different approaches to error correction.

Tools from 2008 or earlier were excluded. Platform-specific tools for 454, Oxford Nanopore and PacBio are only mentioned in the first, second and third row, respectively. The tools listed in the other rows should all be applicable to various data types from different platforms.

**Table 3.** Overview of software implementing the discussed error correction approaches (more details in Supplementary Table S2). This includes software where error correction is only one step of a more complex procedure, such as in software for *de novo* assembly or haplotype reconstruction. Column Q specifies, whether a tool makes use of the base calling quality scores. Column I specifies, whether a tool can correct indels. Values of ‘0.5’ indicate indel correction potential, i.e. that a tool already implicitly corrects some but not all indels or that it could easily be extended to correct them

Tool	Main approach	Data structure(s)	*k*-mer length	k choice reasoning	Global *k*-mer trust threshold	Error model	Correction	Q	I	Further distinctions
Acacia	MSA and clust	Hash of RLE prefixes	6 (RLE!)	–		Emp hopo uc and oc probs	Iterative MSA, iterative clust refinement using statistical tests, clust cons	–	0.5	6-mers of RLE read prefixes; tests all RL discrepancies using 3 bins: uc, main mode, oc; breaks clust w/ consistent mismatches to cons; includes demultiplexing
AHA	Read alignment						Implicit LR corr in scaffolding	–	0.5	Scaffolding of existing contigs from SRs by LRs, initial corr of LRs by short reads
ALLPATHS	*k*-mer spec	–	16, 20 and 24	–	Dyn: 1st loc min of emp *k*-mer cov dist	–	Min Hamming D (Q weighted) corr per read	1	–	Read correct only if all *k*-mers of three different ks solid
ALLPATHS-LG	*k*-mer freq	–	24	Balance uniq versus sensitivity		–	Majority col vote (Q weighted)	1	–	24-mer with 1 base gap in middle (every 13th base corr), contiguous 24-mer: all cols with >6 reads, consistency check of changes within read
AmpliconNoise	Flowgram and corr reads clust (EM)	–				(i) Emp pyroseq errors, (ii) emp PCR errors (learned confusion matrix)	(i) and (ii): EM of mixture model for flowgram/read generation likelihood of obs freq assuming certain generating sequences	–	1	Mixture model with 1 exponential dist component per true seq, learns confusion matrix for PCR error corr from corrected pyroseq reads, aimed at 454 pyrosequencing of amplicons
ARACHNE	MSA	–	8–24			–	Majority col vote (Q weighted), only applied when alt base has strong cov diff	1	1	Chimera detection if central region with decreased cov (= drop in overlaps with other reads)
AutoEdit	MSA and chromatogram	–				Resolvedness of chromatogram peaks over regions	Base recall using base freqs from assembly/mapping	1	1	Base recall, integration of chromatogram context info, aimed at Sanger data
BayesHammer	*k*-mer spec and Hamming graph	Repl sorted *k*-mer lists (Hamming graph), disjoint set (connected components)	21 (def)	–		Error prob = Q	Improves Hammer by subclustering of connected components and read-based correction	1	–	*k*-means clustering of connected components, penalized by Bayesian information criterion; *k*-mer spectrum based on cluster quality; outputs reads
BFC	*k*-mer spec	Bloom filter and hash table	31,55	Trade-off repeat resolution versus *k*-mer coverage	Man: 3	–	Walk through read from longest trusted region, find optimal correction by extension with penalties for corr	1	–	Refined from fermi; exhaustive search of corr, including corr of trusted *k*-mers; skip testing of alternatives for high-quality bases in trusted *k*-mers
BLESS	*k*-mer spec	Hash table (counting), bloom filter (*k*-mer spec)	–	(i) #*k*-mers/4^k ≤ 0.0001 (ii) max # corr bases	Dyn: 1st loc min of emp *k*-mer cov dist	–	Find solid min edit D path between solid *k*-mer islands in reads, paths beyond read ends for errors at ends	1	.5	Reverses some bloom filter false positive changes, better corr of errors at read ends by read extension
Bloocoo	*k*-mer spec	Bloom filter	31 (def)	–	Man (def = 3–6)	–	As Musket	–	.5	As Musket; accepts only corr supported by multiple solid *k*-mers to avoid bloom false pos corr
Blue	*k*-mer spec	Hash table (partitioned)	*k* > 20 (manual)	Uniq (manual)	Dyn per-read thr: 1/3 of harmonic mean of *k*-mer cov, excluding low-cov *k*-mers	Low-cov, cov drops, end of hopo runs	Left-to-right depth-first traversal of all viable *k*-mer corr	–	1	Decouples *k*-mer spec generation from error corr, allowing cross-corr between data sets
Coral	MSA	Hash table (*k*-mer to reads map), adjacency list	k ≈ log_4|G|	Uniq, but k < L/2 (existence of correct *k*-mer in read)		–	Corr w/ thr of Q weighted rel base covs	1	1	Considers MSA Q
CUDA-EC	*k*-mer spec	Counting bloom filter	20	‘Illumina def’	Man: assume Poisson dist of true *k*-mers	–	Min Hamming D corr, max # *k*-mer corr first, greedy per read	1	–	Intro of bloom filter and CUDA parallelization
DecGPU	*k*-mer spec	Bloom filter	21 (def)	–	Man (def = 6)	–	Majority col vote	–	–	CUDA and MPI parallelization
DeNoiser	Flowgram clust (EM)	–				(AmpliconNoise:(i))	(AmpliconNoise:(i))	–	1	Fast pre-clust using exact prefixes, aimed at 454 pyrosequencing of amplicons
ECHO	MSA	Hash table (*k*-mer to reads map), adjacency list	k ≈ L/6	Trade-off discussion		Read pos specific confusion matrix (EM estimated)	Max a posteriori estimate of base call given base covs at col weighted by confusion matrix	–	–	Heterozygosity model
ECTools	MSA	–				–	Corr of LRs with unitigs from pre-assembled SRs	–	1	(i) Unitigs from SRs; (ii) align LRs to unitigs; (iii) optimization using longest increasing subsequence; (iv) correction to unitigs
EDAR	*k*-mer spec and *k*-mer freq clust of read pos	–	–	–	Dyn: 1st loc min of emp *k*-mer cov dist	–	Removes error bases (splits reads)	–	0.5	GC content adjustment (per *k*-mer), identify and remove error bases as pos cluster within read of low *k*-mer freq (variable bandwidth mean-shift method)
EULER	*k*-mer spec	–	20, 100	–	Man: X	–	Min Hamming D corr, max # *k*-mer corr first, greedy per read	–	–	Initial usage of *k*-mer spec for error corr
EULER	*k*-mer spec	–	15–20	Shorter *k*-mers always solid	Man: X	–	Min Levenshtein D corr per read, dyn programming	–	1	
EULER-SR	*k*-mer spec	–	15	–	Man: assume Poisson dist of true *k*-mers	–	Min Hamming D corr, max # *k*-mer corr first, greedy per read	–	–	Iterative relaxation of *k*-mer trust thr (high cov to low)
EULER-USR	*k*-mer spec	Repeat graph (simplified de Bruijn) of read prefixes	20	–	Dyn: 1st loc min of mixed Poisson and Gaussian dist fit	–	Min Hamming D corr, max # *k*-mer corr first, greedy per readprefix, suffix corr w/ repeat graph of prefixes	–	–	
fermi	*k*-mer spec and *k*-mer freq	Suffix array (BWT and FMD index), hash table (*k*-mer spec)	23 (def)	–	Man: def=3	–	Prefix majority vote for low Q base occ	1	–	
Fiona	*k*-mer freq	Suffix array (partial)	Various	As HiTEC	Dyn: from *k* and error prob	Hierarchichal statistical model	Per possible corr: majority vote of all overlapping pos of correct reads, greedy among diff corr pos within read	–	1	Details of statistical model: see Suppl. Table 2
FreClu	Clust reads w/ Hamming D	Hash table, repl sorted read lists (Hamming D)				Read pos and Q specific base confusion matrix	Count corr	1	–	Cluster reads by Hamming distance, create cluster tree from covs, then maps only root read, extends POLYBAYES error model, aimed at RNAseq
Hammer	*k*-mer freq and Hamming graph	Repl sorted *k*-mer lists (Hamming graph), union-find (connected components)	55 (def)	Trade-off discussion		–	Corr of *k*-mers to max likelihood cons of connected component, break likelihood ties by majority vote on Q-weighted *k*-mer covs	1	–	No uniform cov assumption, singletonCutoff for min cov (def = 1), saveCutoff to keep multiple *k*-mers per cluster if cov above, outputs *k*-mers only
HECTOR	k-hopo spec	Bloom filter and hash table	21 (def, k-hopo)	–	Dyn: 1st loc min of emp *k*-mer cov dist	–	As Musket, but using k-hopos to test for base trust	–	1	k-hopos instead of *k*-mers for emp cov dist: k-hopo encodes k consecutive hopo runs by 1-byte RLE; allows up to 3-base indels
HiTEC	*k*-mer freq	Suffix array	Various	Min false negatives or false positives given per-base error rate	Dyn: from |G|, L, N, substitution error prob	–	Unambiguous corr: majority vote, break ties by 2 nt lookahead	–	–	Iterates over various *k*-mer sizes: values either minimize # uncorrectable reads (false negatives) or # destructible reads (false positives)
Hybrid-SHREC	*k*-mer freq	suffix trie	(SHREC)	(SHREC)	Adjusted to current k by tuning parameter alpha	–	(SHREC) plus look up and down one trie level (rerooting for indel corr)	–	1	Inspects multiple k
KEC	*k*-mer spec and *k*-mer freq clust of read pos and MSA	Hash	25 (def)	Clear weak/solid separation versus error resolution	Dyn: end of 1st stretch of 0 cov in emp *k*-mer cov dist	–	Min edits per read in weak regions with max resulting *k*-mer cov	–	1	Identify error regions as in EDAR; iterate over rounds of error corr; eliminate very low-frequency haplotypes from MSA of unique reads; not benchmarked against other *k*-mer corr
Lighter	*k*-mer spec	Bloom filter	23	Max gain in correct bases		–	as BLESS	1	–	Three passes over reads: (i) subsample *k*-mers into 1st bloom filter, (ii) trusted read parts of length k into 2nd bloom filter, (iii) as BLESS
LoRDEC	*k*-mer spec	de Bruijn graph in bloom filters	17–21 (def)	–	Man (def = 2–3)	–	Corr of weak LR regions by traversal of SR de Bruijn graph	–	1	de Bruijn graph from solid *k*-mers of SRs; corr of weak LR regions by graph traversal(s) from solid *k*-mer regions as seeds
LSC	MSA					–	Hybrid read set cons corr of RLE compressed reads	–	1	(i) RLE compress SRs and LRs, (ii) map RLE SRs onto RLE LRs, (iii) correct LRs to MSA cons and trim to SR covered regions
MisEd	MSA	Table (*k*-mers to reads map)				–	Majority vote if col NOT defined nucleotide pos (prob model to test coincidence of deviations)	1	–	Alt base linkage within reads (differentiation repeats/polymorphisms versus errors)
Musket	*k*-mer spec	Bloom filter into hash table	21 (def)	–	Dyn: 1st loc min of emp *k*-mer cov dist	–	Two-sided conservative, one-sided aggressive and voting-based refinement	–	–	Corr unambiguous errors if change makes leftmost and rightmost overlap *k*-mer solid, otherwise extension from solid region and col majority vote plus lookahead *k*-mer consistency check (def = 2) plus max changes per *k*-mer (def = 4)
MyHybrid	MSA	suffix array	k ≈ log_4(200*|G|)	(Quake)	Dyn: half the exp *k*-mer occs given error prob	–	Majority col vote	–	1	*k*-mer cov thr for MSA seeds
N-corr	Hopo pattern in 454 reads	–				Hopo pattern	–	–	–	
Nanocorr	MSA	–				–	Cons of SR alignment	–	1	Align SRs to LRs, dynamically find optimal alignment
pacbio_qc	Read filter (SVM)	See LIBSVM package				Mean Q and CCS pass # of read	SVM regression trained on known spike-in	1		
PBcR	MSA	–					Hybrid read set corr	–	0.5	(i) Map SRs onto LRs, (ii) correct LRs to MSA cons
Potts model	Reads clust w/ Hamming D	–				Error prob = Q	Max likelihood Potts model estimation on Hamming neighbourhoods by clust col pos	1	–	
PREMIER	*k*-mer freq (HMM, EM)	–	Optimize performance	Uniq versus sufficient cov		*k*-mer transition probs from EM of its read pos, Q, cov and a base confusion matrix	Max likelihood of seq generation w/ HMM	1	–	
proovread	Read mapping	Alignment matrix per LR				Mapping w/ penalties portraying error probs	Cons calling by majority vote		1	Penalties for mapping reflect PacBio error probs; quality scores from SR base support; chimera detection; iterative w/ increasing sensitivity
PSAEC	*k*-mer freq	Suffix array (partial)	20 (ex)	–	Dyn: from |G|, L, N, substitution error prob	–	(HiTEC)	–	–	Runtime and memory optimization by using only partial suffix array
PyroNoise	Flowgram clust					Emp pyroseq errors	EM of mixture model for flowgram generation likelihood of obs freq assuming certain generating sequences			
Quake	*k*-mer spec	Bit array index (*k*-mer spec)	k ≈ log_4(200*|G|)	Prob = 0.01 for *k*-mer occ in *k*-mer space 4^k/2	Dyn: mixed dist model fit and likelihood ratio	Q specific confusion matrix (learned from unambiguous initial corrections)	Max likelihood *k*-mer changes, min Q pot error pos 1st	1	–	1st proper discussion of *k*-mer length trade-off, sophisticated *k*-mer dist mixture model: weak = Gamma, solid = Poisson, repeat *k*-mer multiplicity = Zeta
QuorUM	*k*-mer freq	–	24 (def)	–		–	Trim if no alt base with cov, correct if only one alt base with cov, one-pos lookahead if multiple alts with non-zero cov, break ties by cov continuity check	1	–	No uniform cov assumption: correcting *k*-mers at sudden cov drop along read, can remove contaminant *k*-mers, does trimming, conservative thr to not inspect high cov *k*-mers
RACER	*k*-mer spec	Hash table	‘Computed from |G|’	–	Dyn: from |G|	–	Unambiguous weak to solid corrs	–	–	
RECOUNT	Read counts (EM)	–				Average of Q values in alignment col gives read and pos specific error prob	Count corr	1		EM of exp read counts
REDEEM	*k*-mer spec	Sparsehash	11 (def)	Uniq of non-repeat *k*-mers	Man (def = 20), BUT dyn freq counts	Learned sparsified *k*-mer confusion matrix	Set each read pos to nt with max prob over all covering *k*-mers, corr of reads with weak *k*-mers	–	–	Compute exp *k*-mer freq given obs *k*-mer freq (EM of max likelihood using *k*-mer misread probs)
Reptile	*k*-mer freq and Hamming graph	Repl sorted *k*-mer list (Hamming graph)	k ≈ log_4|G| or 10 ≤ k ≤ 16	Uniq of average *k*-mer	Man: high and medium confidence *k*-mer thrs (Q)	–	Min Hamming D corr of two consecutive *k*-mers (a tile) at a time if tile has low cov and low Q pos	1	–	*k*-mer freq ratio thr applied to pot correct versus pot error *k*-mers in correction step (def=2)
SEECER	MSA, col clust and HMM	Hash table (*k*-mers to reads map)	17 (def)			Learn HMM parameters for each MSA contig	One HMM per contig, read assignments to HMMs by log likelihood from Viterbi's alg	–	1	Separates polymorphisms from errors by spec clust and spec relaxation of *k*-means on mismatch cols, aimed at RNAseq data
SGA	*k*-mer freq	Suffix array (BWT and FM index)	31 (def)	Emp choice from error corr on read subset with various (‘sga stats’)	Dyn: loc min of emp *k*-mer dist	–	Leftmost and rightmost overlap *k*-mer check for alt bases with cov above thr	1	–	Optional to use only base pos of PHRED Q above 20 for *k*-mer counting and thr determination
SGA	MSA	Suffix array (BWT and FM index)				–	Corr conflicting cols if single base above corr cov thr (def=3)	1	–	Checks mismatch linkage (conflict cols of read = multiple alt bases above conflict cov thr; exclude reads with mismatch at all conflicts from read's corr MSA)
ShoRAH	MSA and read clust	Hash tables				–	Loc majority rule within haplotype (in three windows overlapping each seq pos)	–	1	Clust reads into haplotypes with Gibbs sampler of posterior dist of Dirichlet process mixture that models haplotypes
SHREC	*k*-mer freq	Suffix trie	[min {log_4(|G|), log_4(n)} + q] ≤ k ≤ s	n = #reads, q: (1/4)^q < p, s: low cov thr for trie level	Adjusted to current k by tuning parameter x	–	Reroot branch if actual node cov below exp node cov (±SD*tuning param) for current trie level	–	–	Inspects multiple k
SleepEC	Read freq and Hamming graph	Lists of nodes, node properties, node links	Read length			Read pos specific base confusion matrix	Stat test of actual read abundance versus predicted	–	–	For RNAseq reads; builds Hamming graph, learn base substitution matrix from trusted connected components
SOAPdenovo	*k*-mer spec	Hash table (*k*-mer freq)	k ≈ log_4|G|	Uniq of average *k*-mer	Man	–	Min Hamming D corr per read by extending high-cov region, majority col vote, dyn programming	–	–	
SOAPdenovo2	*k*-mer spec	Hash or index table	k ≈ log_4(20*|G|)	*k*-mer space >10x genome *k*-mer space	Man	–	Col voting on unambiguous errors, voting on possible change paths (rooted at correct pos) for ambiguous errors	–	–	
SysCall	MSA col classifier (logistic regression)	Matrix (rows = pot het pos, cols = features)				Posterior prob based on (i) Q diff to pos neighbours, (ii) seq context, (iii) strand bias	Logistic regression model, distinguish errors from het at each pot het pos (thr 0.5)	1	–	
Trowel	*k*-mer spec	Hash table (*k*-mer as key, max Q of corr pos as entry)	As Quake	As Quake	–	Error prob = Q	Compare Musket: (i) gapped *k*-mer (brick) correction, (ii) one-sided *k*-mer correction for read ends and consecutive error pos	1	–	*k*-mer spec purely Q-based (global Q thr), iteratively expanded by read-based boosting of low-Q pos

Abbreviations: # = number of, alt = alternative, alg = algorithm, BWT = Burrows Wheeler transform, CCS = circular consensus sequence, clust = cluster(ing), col = column, cons = consensus, corr = correction, cov = coverage, D = distance, def = default, diff = difference, dist = distribution, dyn = dynamic, EM = expectation maximization, emp = empirical, exp = expected, FM = Ferragina Manzini, freq = frequency, |G| = genome size, HMM = hidden Markov model, hopo = homopolymer, I = indel = insertion/deletion, k = *k*-mer length, L = read length, LR = long read, loc = local, man = manual, max = maxmimum, min = minimum, MSA = multiple sequence alignment, N = #reads, na = not available, obs = observed, oc = overcall, occ = occurrence, pos = position, pot = potential, prob = probabilty, Q = quality, rel = relative, repl = replicated, RLE = run-length encoding, spec = spectrum, SR = short read, SVM = support vector machine, thr = threshold, uc = undercall, uniq = uniqueness, X = experience.

**Table 4.** Citations and software URLs of error correction tools

Tool	Author, year	Citation	Software URL
Acacia	(Bragg *et al.*, 2012)	[88]	http://sourceforge.net/projects/acaciaerrorcorr/
AHA	(Bashir *et al.*, 2012)	[96]	https://github.com/PacificBiosciences/Bioinformatics-Training/wiki/AHA
ALLPATHS	(Butler *et al.*, 2008)	[69]	http://www.broadinstitute.org/science/programs/genome-biology/computational-rd/computational-research-and-development
ALLPATHS-LG	(Gnerre *et al.*, 2011)	[77]	http://www.broadinstitute.org/software/allpaths-lg/blog/?page_id=12
AmpliconNoise	(Quince *et al.*, 2011)	[91]	https://code.google.com/p/ampliconnoise/downloads/list
ARACHNE	(Batzoglou, 2002)	[40]	http://www.broadinstitute.org/science/programs/genome-biology/computational-rd/computational-research-and-development
AutoEdit	(Gajer, 2004)	[80]	not available (any more)
BayesHammer	(Nikolenko *et al.*, 2013)	[60]	http://bioinf.spbau.ru/en/spades
BFC	(Li, 2015)	[71]	https://github.com/lh3/bfc
BLESS	(Heo *et al.*, 2014)	[104]	http://sourceforge.net/projects/bless-ec/
Bloocoo	(Drezen *et al.*, 2014)	[105]	https://gatb.inria.fr/gatb/binaries/
Blue	(Greenfield *et al.*, 2014)	[64]	http://www.bioinformatics.csiro.au/blue
Coral	(Salmela and Schroder, 2011)	[61]	http://www.cs.helsinki.fi/u/lmsalmel/coral/
CUDA-EC	(Shi *et al.*, 2010a, 2010b)	[106, 107]	http://sourceforge.net/projects/cuda-ec/
DecGPU	(Liu *et al.*, 2011)	[108]	http://decgpu.sourceforge.net/homepage.htm#latest
DeNoiser	(Reeder and Knight, 2010)	[90]	http://www.microbio.me/denoiser/
ECHO	(Kao *et al.*, 2011)	[62]	http://uc-echo.sourceforge.net/
ECTools	(Lee *et al.*, 2014)	[99]	https://github.com/jgurtowski/ectools
EDAR	(Zhao *et al.*, 2010)	[75]	not available
EULER	(Pevzner *et al.*, 2001)	[45]	http://cseweb.ucsd.edu/∼ppevzner/software.html
EULER	(Chaisson *et al.*, 2004)	[65]	http://cseweb.ucsd.edu/∼ppevzner/software.html
EULER-SR	(Chaisson and Pevzner, 2008)	[68]	http://cseweb.ucsd.edu/∼ppevzner/software.html
EULER-USR	(Chaisson *et al.*, 2009)	[83]	http://cseweb.ucsd.edu/∼ppevzner/software.html
fermi	(Li, 2012)	[55]	https://github.com/lh3/fermi
Fiona	(Schulz *et al.*, 2014)	[52]	http://www.seqan.de/projects/fiona/
FreClu	(Qu *et al.*, 2009)	[21]	http://mlab.cb.k.u-tokyo.ac.jp/∼quwei/DeNovoShortReadclust/
Hammer	(Medvedev *et al.*, 2011)	[59]	http://bix.ucsd.edu/projects/hammer/
HECTOR	(Wirawan *et al.*, 2014)	[92]	http://hector454.sourceforge.net/
HiTEC	(Ilie *et al.*, 2011)	[49]	http://www.csd.uwo.ca/∼ilie/HiTEC/
Hybrid-SHREC	(Salmela, 2010)	[47]	http://www.cs.helsinki.fi/u/lmsalmel/hybrid-shrec/
KEC	(Skums *et al.*, 2012)	[93]	http://alan.cs.gsu.edu/NGS/?q=content/pyrosequencing-error-correction-algorithm
Lighter	(Song *et al.*, 2014)	[109]	https://github.com/mourisl/Lighter
LoRDEC	(Salmela and Rivals, 2014)	[85]	http://atgc.lirmm.fr/lordec/
LSC	(Au *et al.*, 2012)	[97]	http://www.healthcare.uiowa.edu/labs/au/LSC/
MisEd	(Tammi, 2003)	[86]	not available
Musket	(Liu *et al.*, 2013)	[110]	http://musket.sourceforge.net/homepage.htm#latest
MyHybrid	(Zhao *et al.*, 2011a)	[43]	not available
N-corr	(Shin and Park, 2014)	[111]	http://nar.oxfordjournals.org/content/suppl/2014/01/27/gku070.DC1/nar-00508-met-k-2013-File010.docx
Nanocorr	(Goodwin *et al.*, 2015)	[101]	https://github.com/jgurtowski/nanocorr
pacbio_qc	(Jiao, 2013)	[28]	http://david.abcc.ncifcrf.gov/manuscripts/pacbio_qc/
PBcR	(Koren *et al.*, 2012, 2013)	[26, 95]	http://cbcb.umd.edu/software/PBcR/
Potts model	(Aita *et al.*, 2013)	[63]	not available
PREMIER	(Yin *et al.*, 2013)	[79]	not available
proovread	(Hackl *et al.*, 2014)	[98]	http://proovread.bioapps.biozentrum.uni-wuerzburg.de/
PSAEC	(Zhao *et al.*, 2011b)	[51]	not available
PyroNoise	(Quince *et al.*, 2009)	[89]	http://userweb.eng.gla.ac.uk/christopher.quince/Software/PyroNoise.html
Quake	(Kelley *et al.*, 2010)	[70]	http://www.cbcb.umd.edu/software/quake/
QuorUM	(Marçais *et al.*, 2013)	[76]	http://www.genome.umd.edu/quorum.html
RACER	(Ilie and Molnar, 2013)	[112]	http://www.csd.uwo.ca/∼ilie/RACER/
RECOUNT	(Wijaya *et al.*, 2009)	[81]	not available (any more)
REDEEM	(Yang *et al.*, 2011)	[113]	http://aluru-sun.ece.iastate.edu/doku.php?id=redeem
Reptile	(Yang *et al.*, 2010)	[58]	http://aluru-sun.ece.iastate.edu/doku.php?id=reptile
SEECER	(Le *et al.*, 2013)	[78]	http://sb.cs.cmu.edu/seecer/
SGA	(Simpson and Durbin, 2012)	[44]	https://github.com/jts/sga
ShoRAH	(Zagordi *et al.*, 2010a, 2011)	[41, 42]	http://www.bsse.ethz.ch/cbg/software/shorah
SHREC	(Schroder *et al.*, 2009)	[46]	http://sourceforge.net/projects/shrec-ec/
SleepEC	(Sleep *et al.*, 2013)	[73]	https://ep.unisa.edu.au/view/view.php?id=46870
SOAPdenovo	(Li *et al.*, 2010)	[66]	http://soap.genomics.org.cn/soapdenovo.html
SOAPdenovo2	(Luo *et al.*, 2012)	[67]	http://soap.genomics.org.cn/soapdenovo.html
SysCall	(Meacham *et al.*, 2011)	[82]	http://bio.math.berkeley.edu/SysCall/
Trowel	(Lim *et al.*, 2014)	[74]	http://sourceforge.net/projects/trowel-ec/

In general, only few tools take into account (some of) the here presented knowledge about errors, their biases, coverage biases and their dependency on the particular sequencing platform. But more specific approaches would improve the accuracy of error correction and thereby improve downstream analyses: read mapping and *de novo* assembly would become more tractable computationally and would give better results, variant calls derived from such alignments would be more accurate. However, higher specificity will potentially limit the scope of a tool to a certain platform or a type of data set. Instead, bearing the six aspects that we just discussed in mind, we believe that future efforts should aim at a whole error correction toolkit, that should be freely available for any use and open-source to encourage distributed development, as is e.g. being developed for variant discovery by the Genome Analysis Toolkit (GATK; [114]) or for sequencing data analysis in general by SeqAn [115], a library kit that the error corrector Fiona is built upon. Depending on the data set at hand, such a toolkit should optimally be able to optionally consider indels, to give a choice whether to assume an even coverage to reduce computational load, or to avoid this assumption, to have platform-specific error models to choose from (or even learn parameters of the model from the data set), to allow using prior information on the expected haplotype and repeat composition of the queried sequence(s) if available or be able to infer haplotypes and repeats from the data itself and to be flexible in the use of one of the three major approaches, depending on which will be most efficient computationally for the combination of requirements of a particular data set. This suggests a modular approach to error correction, similar to the above mentioned GATK and SeqAn and as software tools in other fields (e.g. variant discovery) also employ error models, individual modules of such a toolkit will be useful beyond stand-alone error correctors and assembly pipelines.

We believe, that the overview and comparison of error correction approaches and tools given here will inform both users and developers and will enable the community: to pursue the proposed modular development of error correction software in the future, to use such a modular approach to flexibly combine ideas from different error correction approaches (as has been recently begun for PacBio, Oxford Nanopore and 454 error correction), to use more of the knowledge about errors and their biases in development in this field and beyond (e.g. for SNV calling) and to create meaningful comparative benchmarks of the here listed tools on comprehensive and representative data sets for many different data types and analysis set-ups.

**Key Points**

- Each sequencing platform has a distinct error profile that relates to the respective sequencing technology. This includes substantial undercoverage of extreme GC contents in all platforms except the PacBio, with Ion Torrent showing the strongest bias. Regarding single nucleotide errors, Illumina and Complete Genomics show more substitution errors than indel errors, for all other platforms it is the other way around. Overall, the short Illumina reads have the lowest error rates, while the long single molecule sequencing reads of PacBio and Oxford Nanopore have the highest error rates. However, PacBio errors appear to be genuinely random, while especially Illumina errors show specific biases, e.g. owing to certain sequence motifs.
- The earliest approach to explicitly address error correction in raw read data, with the intention of facilitating a *de novo* assembly of them, is based on the *k*-mer spectrum. The idea is to create a list of trusted *k*-length sequences (*k*-mers) from the read data and to correct all untrusted *k*-mers towards this spectrum, where trusting a *k*-mer usually requires a certain minimum coverage. The most important parameters that need to be determined for this method are the *k*-mer length and the coverage threshold. For both, there is no choice that works best in all instances and newer tools try to completely avoid a global coverage threshold to be applicable to data with non-uniform coverage across the queried sequence. Major performance improvements have recently been achieved by using bloom filters to store the *k*-mer spectrum and related information.
- Another early approach to error correction that is closely connected to *de novo* assembly, was to generate MSAs of all reads and to correct to a consensus. However, the initial all-versus-all alignment approach became intractable with the read numbers of high throughput next generation sequencing and was only later adapted using intelligent indexes of possible read overlaps to seed the alignments (e.g. using a *k*-mer index or read clustering techniques). In general, MSA-based methods are usually well-suited for the resolution of indel errors, do not require a global frequency threshold or the choice of a *k*-mer length and can easily integrate read context information in the correction decision (e.g. repeat disambiguation or haplotype linkage). Recently, they have become more popular with the advent of single molecule sequencing technologies that produce fewer but longer reads.
- The third major approach is to use a suffix array (or derivatives thereof) for error correction. The sorted list of all possible suffixes of all reads in a data set can not only be used to seed a MSA, but can also itself be queried efficiently to provide coverage values for *k*-mers of any possible length. In effect this yields a more flexible version of the *k*-mer spectrum approach and recent implementations using the BWT and the FM index have made this approach tractable for high throughput data sets. It is noteworthy, that the respective tools were among the first to employ statistical models of errors in the sequencing process and to automatically choose and check multiple *k*-mer lengths for error decisions.
- In the past years, error and repeat models have evolved significantly. Whereas earlier tools only worked with uniform error rates, newer tools often use confusion matrices for substitution errors, that can be conditional on quality scores and read position, and some employ separate insertion and deletion rates. Such values are either known for the platform or can be approximated from the respective data set under consideration. Also, tools have begun to explicitly model the sequencing process in a probabilistic manner. In parallel, models have tried to account for repeats or haplotypes in the queried sequence(s): by using particular data structures; by looking at variation linkage within reads; or by estimating haplotypes probabilistically.
- Only two studies have so far provided benchmarks of a subset of the here presented tools. As a result, most use cases of an error corrector do not allow an informed tool choice without performing a specific benchmark and especially end-users of assemblers mostly rely on the error corrector built into their software of choice. The high-throughput sequencing community would greatly benefit from specific comparative benchmarks on comprehensive data sets. These should represent different data types and use cases. The methodological overview provided here can inform the choice of tools to benchmark for a particular set-up.

## Supplementary data

Supplementary data are available online at http://bib.oxfordjournals.org/. This includes detailed tables, supplementary notes and vector graphic versions of all figures in this publication.

Supplementary Data
