# Supplementary material for: Denoising DNA deep sequencing data—high-throughput sequencing errors and their correction
Source: Brief Bioinform. 2015 May 29;17(1):154–79. doi: 10.1093/bib/bbv029 (PMC4719071; doi:10.1093/bib/bbv029)
Supplement: Supplementary Data [file supp_17_1_154__index.html]

Denoising DNA deep sequencing data—high-throughput sequencing errors and their correction — Supplementary Data 

# Denoising DNA deep sequencing data—high-throughput sequencing errors and their correction

## Supplementary Data

files

- Supplementary Data - zip file
