# Supplementary material for: Denoising DNA deep sequencing data—high-throughput sequencing errors and their correction
Source: Brief Bioinform. 2015 May 29;17(1):154–79. doi: 10.1093/bib/bbv029 (PMC4719071; doi:10.1093/bib/bbv029)
Supplement: Supplementary Data [file supp_bbv029_suppl_data_updated.zip › Supplementary_updated.pdf]

Supplementary Notes and Vector Graphics

1 Supplementary Notes

Supplementary Note 1: MSA-based error correction tools

The Arachne assembler [39,40] was the first tool to use a multiple sequence alignment (MSA) approach for read error correction. By using a table of *k*-mer occurrences across all reads to decide which reads to align with each other (i.e. only reads having an exact overlap of length *k*, grey dashed arrows and table in Figure 3), a MSA was constructed from comparably few pairwise alignments (in this case simply creating an assembly). MisEd [86] made this approach more flexible by using inexact matches as seeds (effectively enabling the correct alignment of reads with errors within seeds). Arachne2 [116] optimised memory usage by reordering the *k*-mer index systematically. However, even these optimisations did not scale to the short read high throughput technologies, leading to two lines of development: most tools abandoned MSAs and focused on *k*-mer counting (Section "k-mer frequencies and spectrum" and Supplementary Note 2), while others optimised the initial step of reducing the number of alignments necessary for the construction of the MSA: (i) the tools ECHO [62], Coral [61] and SEECER [78] make the *k*-mer based pre-screening more efficient by storing their *k*-mer index in a hash; (ii) ShoRAH [41,42] initially does a reference mapping and then constructs a MSA from that; and (iii) MyHybrid [43] and SGA [44] do the pre-screening using a suffix array or a derivative thereof, respectively (Section "Suffix tries and arrays, the Burrows Wheeler transform (BWT) and the Full-text index in Minute space (FM index)").

Supplementary Note 2: Implementations of the k-mer Spectrum

In EULER, the *k*-mer spectrum approach was initially implemented as an iterative procedure [45], then improved using dynamic programming [65] and further accelerated to scale to high-throughput technologies with short reads (EULER-SR [68]; EULER-USR [83]).

Also, space requirements for the *k*-mer spectrum hash table can be reduced by an approach introduced in CUDA-EC [106 ,117]: the probabilistic Bloom filter [118] for *k*-mer counting and spectrum membership checks. The Bloom filter is also used in DecGPU [108], Musket [110], BLESS [104], Bloocoo [105] and Lighter [109]. And two interesting recent uses of the Bloom filter are HECTOR [92], which uses it to efficiently store a homopolymer spectrum instead of a *k*-mer spectrum, and LoRDEC [85], which uses it to efficiently store a de Bruijn graph of short reads, implementing the ideas of Chikhi and Rizk [119], and Salikhov *et al.* [120].

Four recent variations of the *k*-mer spectrum approach are also noteworthy:

(i) In Blue [64], the generation of the *k*-mer spectrum is uncoupled from the error correction.

I.e., the tool can generate the *k*-mer spectrum on one dataset (e.g. short reads with a lower error rate) and use it to correct another dataset generated from the same sample (e.g. long reads with a higher error rate; see also the hybrid error correction in Section "Platform specific error correction").

(ii) BayesHammer [60] introduced a major modification of the k-mer spectrum. Instead of constructing it from *k*-mer frequencies with a global threshold, it first clusters *k*-mers using their Hamming distance and quality scores (Sections "Substitutions only vs. substitutions plus indels: Hamming vs. Levenshtein distance" and "Removing the uniformity of coverage assumption") and then uses the most abundant *k*-mers from high quality clusters as the trusted (or solid) *k*-mers. In addition, it extends the spectrum by finding high-quality reads, in which all bases are covered by at least one solid *k*-mer. Any non-solid *k*-mers from such reads are subsequently marked as solid.

(iii) Trowel [74] only includes *k*-mers in its trusted spectrum, if all bases in it are within the top 8% of the data set's quality values in at least one read. As in BayesHammer, the *k*-mer spectrum is not static, but is iteratively expanded during the read centered correction: with each correction, the quality of the corrected base is "boosted" to the maximum quality of the supporting (gapped) *k*-mer and if this boosting results in any new solid *k*-mers, these are added to the *k*-mer spectrum.

(iv) Lighter [109] creates its spectrum of trusted *k*-mers in two steps: In a first pass over all reads, it includes any *k*-mer it encounters into a first bloom filter with a specified probability alpha. In a second pass it then determines whether a read position is trusted by two criteria: Its quality score must be above the minimal fifth percentile of both the quality score distributions of the first and the last positions of the first one million reads in the dataset and the read position must be covered by a minimum number of *k*-mers in the first bloom filter, with this coverage dependent on the positions proximity to the closest read end. All consecutive stretches of trusted read positions with length *k* are then included in the second bloom filter, creating the trusted *k*-mer spectrum for the subsequent correction.

Supplementary Note 3: Varying k-mer lengths in suffix tries and arrays

Suffix tries and arrays sort and thus group suffixes of varying length lexicographically (Figures 5 and 6). The respective data structures therefore natively support the inspection of several *k*-mer lengths by either looking at different levels of the tree or by analysing prefixes (of sorted suffixes) of varying length. (Hybrid) SHREC [46,47] only inspects node weights at intermediate levels of the tree -- corresponding to intermediate *k*-mer lengths -- and gives clear boundaries: the lower bound is the smaller value out of log_4(|*G*|) (which ensures *k*-mer uniqueness in the reference) and log_4(*n*) (*n* is the read count, which is lower than |*G*| if there are fewer reads than positions in *G*), both augmented by a q < log_(1/4)*p* (*p* is the sequencing error rate); the

upper bound for k is the point where edge weights go beneath a threshold that signifies that the coverage is too low. HiTEC [49] determines two values of k: the first one minimizes the probability of uncorrectable reads (false negatives), the second one the probability of destructible reads (false positives). These calculations are based on the expected number of correct and erroneous k-mers at a certain position, given a per base error rate estimate. Iterations of the correction procedure then vary k from one optimum to the other and explore several values of k around each of the optima in the various runs of the correction procedure. Fiona [52] extends this model, making it usable on datasets with variable read lengths, and further optimises corrections: it not only considers one k-mer length at a time, but maximizes coverage support from correct read overlaps from all inspected k-mer lengths and to both sides of each error position; and, within each read, it corrects in a coordinated fashion, by starting with the maximally supported correction in a read and only adding further corrections if they do not conflict with the overlaps from supporting read of previous corrections.

Supplementary Note 4: Flavours of global k-mer frequency trust thresholds

The ALLPATHS assembler [69] was the first tool to automatically choose a k-mer frequency trust threshold from the empirical k-mer distribution. It did so by finding the first local minimum between the peaks of two assumed sub-distributions for the erroneous and the correct k-mers (Figure 7). Most of the other tools subsequently opted for a similar strategy to determine which k-mers to trust and some added further refinements: Reptile [58] computes a separate frequency distribution for k-mers and quality values and then asks the user to derive a threshold for each from the respective plots. EULER-USR [83] explicitly fits a mixture model to the empirical k-mer distributions (with the untrusted k-mers modelled as a Poisson distribution and the trusted k-mers as a Gaussian distribution) and then takes the first local minimum of this model as the k-mer frequency threshold. Quake [70] weights k-mer frequencies by quality values to more clearly separate distributions, resulting in continuous "q-mer counts". To accommodate for these continuous values, the authors substitute the Poisson distribution for untrusted k-mers with a Gamma distribution. In addition, they consider the heavy tail of high multiplicity q-mers to result from repeats in the queried sequence (Figure 7): they model them as a Zeta distribution and then project the respective q-mer counts into the Gaussian distribution for correct q-mers by sampling a copy number from the Zeta distribution. Using the maximum likelihood fit of this mixture model, Quake then determines the q-mer frequency threshold. REDEEM [113], on the other hand, does not eliminated the fixed global threshold (default is 20, even though a method for dynamically determining this is also given by the authors), but instead focuses only on refining an expected (and thus more flexible) k-mer frequency given the observed one. To this end, REDEEM learns k-mer misread probabilities from a control lane in the sequencing experiment (or uses known technology defaults; see also Section "Denoising with statistical error models" and Supplementary Note 5)

and then uses these in an expectation maximization procedure to find the frequency with maximum likelihood for each *k*-mer. Finally, the global threshold is then applied to these maximum likelihood frequencies. In SGA [44], a much simpler addition to threshold finding is made: the user can require a higher coverage for low-quality *k*-mers (quality below a phred score of 20). And HECTOR [92] does not determine a *k*-mer coverage threshold, but a *k*-hopo threshold: (i) it represents 454 pyrosequencing reads, that are especially prone to homopolymer length errors, by their run-length encoding (e.g AAATGG as the pair sequence (3,A)(1,T)(2,G)); (ii) it creates *k*-hopos of length *k* from these (for *k*=2 our example would give [(3,A)(1,T)] and [(1,T)(2,G)]); (iii) it generates a coverage histogram for these *k*-hopos -- which gives a bimodal distribution that would not be found for the *k*-mer coverage of 454 reads -- and takes the first local minimum as the *k*-hopo threshold.

Supplementary Note 5: Base confusion matrices in error models

The probability that a certain base is erroneously mistaken for another base can be strongly biased by the sequencing platform, but also by each individual sequencing run or by preparation steps. Instead of assuming a uniform base confusion probability, several tools therefore determine base confusion matrices (recording all possible confusions). We here survey them in a roughly chronological order of tool publication:

FreClu [21] learned the substitution probabilities of Illumina data from reads from a known BAC sequence. It derives confusion matrices that depend on a base's quality value and its position within the read. I.e. it produces a separate confusion matrix for every possible combination of quality value and read position from the training data set.

Quake [70] also uses quality specific (but not read position specific) confusion matrices, but learns them from the current dataset by counting the substitutions made in an initial round of unambiguous corrections. It smooths the estimates across the quality values with a Gaussian kernel.

AmpliconNoise [91], a tool aimed at 454 pyrosequencing of amplicons, applies two separate correction steps -- first removing noise from pyrosequencing and in a second step removing errors from the pre-amplification PCR -- and uses a base confusion matrix in the PCR error correction. Its matrix is derived from a control run with known sequences.

REDEEM [113] uses a slightly different matrix: a *k*-mer confusion matrix that it learned from the data. As 4^*k* possible *k*-mers would produce a very large confusion matrix, it only considers existing *k*-mers and further sparsifies the matrix by only considering confusions between very similar *k*-mers (within a certain Hamming distance).

ECHO [62] uses an expectation maximization (EM) algorithm based on its probabilistic model to estimate a base confusion matrix for every possible base position within a read.

PREMIER [79] also uses an EM algorithm to estimate confusion probabilities, albeit only as one parameter of several in a hidden Markov model (HMM) described in more detail in Section “Denoising with statistical error models“.

An error correction tool by Sleep *et al.* [73] creates a Hamming graph (Sections “Substitutions only vs. substitutions plus indels: Hamming vs. Levenshtein distance“ and “Removing the uniformity of coverage assumption“; Figure 4D) from RNAseq reads (Hamming distance of 1) and learns the read position specific base confusion matrix from high confidence connected components in the graph.

Supplementary Note 6: Hierarchical statistical model of Fiona

In Fiona [52], error correction decisions are based on a hierarchical statistical model: Assuming a uniform coverage, it models the expected *k*-mer coverage as a Poisson distribution with its parameter determined from the approximate genome length, the *k*-mer length, the average read length and the total number of reads. Using this parameter and additionally assuming a uniform error probability, the parameter of another Poisson distribution is determined: it models the expected coverage of a *k*-mer possessing a certain number *i* of errors. Together with the expected proportion of reads containing exactly *i* errors -- which can be calculated for each *i* -- this expected coverage of a *k*-mer with *i* errors is used to calculate the probability of an actually observed *k*-mer coverage (conditional on the number of errors *i)*. This probability is summed for all possible error containing *k*-mers generated from this k-mer (i>0) and the probability of the error free *k*-mer (i=0) is calculated separately. Then, the log odds ratio of these probabilities for error containing *k*-mers to error free *k*-mers (given the observed *k*-mer coverages) is taken. Also, a second log odds ratio is calculated: the general probability that any *k*-mer of length *k* contains errors vs. no errors. If the log odds ratio of an error given the observed *k*-mer coverage of a certain *k*-mer is higher than the log odds ratio of a *k*-mer error in general, the *k*-mer is considered erroneous.

Supplementary Note 7: Contig HMMs for error correction

SEECER [78] uses per-position hidden Markov models (HMMs) to represent the sequencing process with errors (Figure 1 in [78]). It first creates initial contigs by identifying reads that have *k*-mer overlaps to a starting read. Each contig is refined by separating coherently different subsets of reads (representing either inexact repeats or different haplotypes) into contigs of their own. One profile HMM [121] per contig -- called a contig HMM -- is then estimated from the contig by either an EM algorithm or a simple scan of the alignment. Contigs are then broken at columns of high entropy in the calculated emission probabilities. Subsequently, the contig and the profile HMM are repeatedly extended and updated by finding further *k*-mer overlaps, repeating the mismatch column clustering and the emission probability

entropy check until no further overlaps can be found. Eventually, reads in the contig are realigned to the contig HMM and corrected towards it, if the likelihood of them being generated by that contig HMM exceeds a given threshold.

Supplementary Bibliography

116. Jaffe DB, Butler J, Gnerre S, *et al.* Whole-Genome Sequence Assembly for Mammalian Genomes: Arachne 2. *Genome Res.* 2003; **13**:91–96

117. Shi H, Schmidt B, Liu W, *et al.* Accelerating error correction in high-throughput short-read DNA sequencing data with CUDA. In: *IEEE Int. Symp. Parallel Distrib. Process., 2009 IPDPS*, 2009; pp. 1–8

118. Bloom BH. Space/Time Trade-offs in Hash Coding with Allowable Errors. *Commun ACM* 1970; **13**:422–426

119. Chikhi R, Rizk G. Space-efficient and exact de Bruijn graph representation based on a Bloom filter. *Algorithms Mol. Biol.* 2013; **8**:22

120. Salikhov K, Sacomoto G, Kucherov G. Using cascading Bloom filters to improve the memory usage for de Brujin graphs. *Algorithms Mol. Biol.* 2014; **9**:2

121. Eddy SR. Profile hidden Markov models. *Bioinformatics* 1998; **14**:755–763

2 Vector Graphics

This section embeds all of the publication's figures as vector graphics for practical reuse, each one preceded by its caption. As part of this Open Access article, they are licensed by Briefings in Bioinformatics as CC-BY 3.0 (creative commons license requiring attribution of the source and indication of changes made: http://creativecommons.org/licenses/by/3.0/).

Figure 1

Sequencing coverage across different local GC contents in three microbes (*P. falciparum*, *E. coli* and *R. sphaeroides*) and a human genome. The bottom panels show the relative fraction of 100-base windows in the respective genome having a certain GC content. The top panels show the relative sequencing coverage for 100-base windows with a certain GC content compared to the respective platform sample's average.

This figure is aggregated and adapted from figures 2 and 3 in [13], according to the Creative Commons Attribution license CC-BY 2.0 (http://creativecommons.org/licenses/by/2.0/).

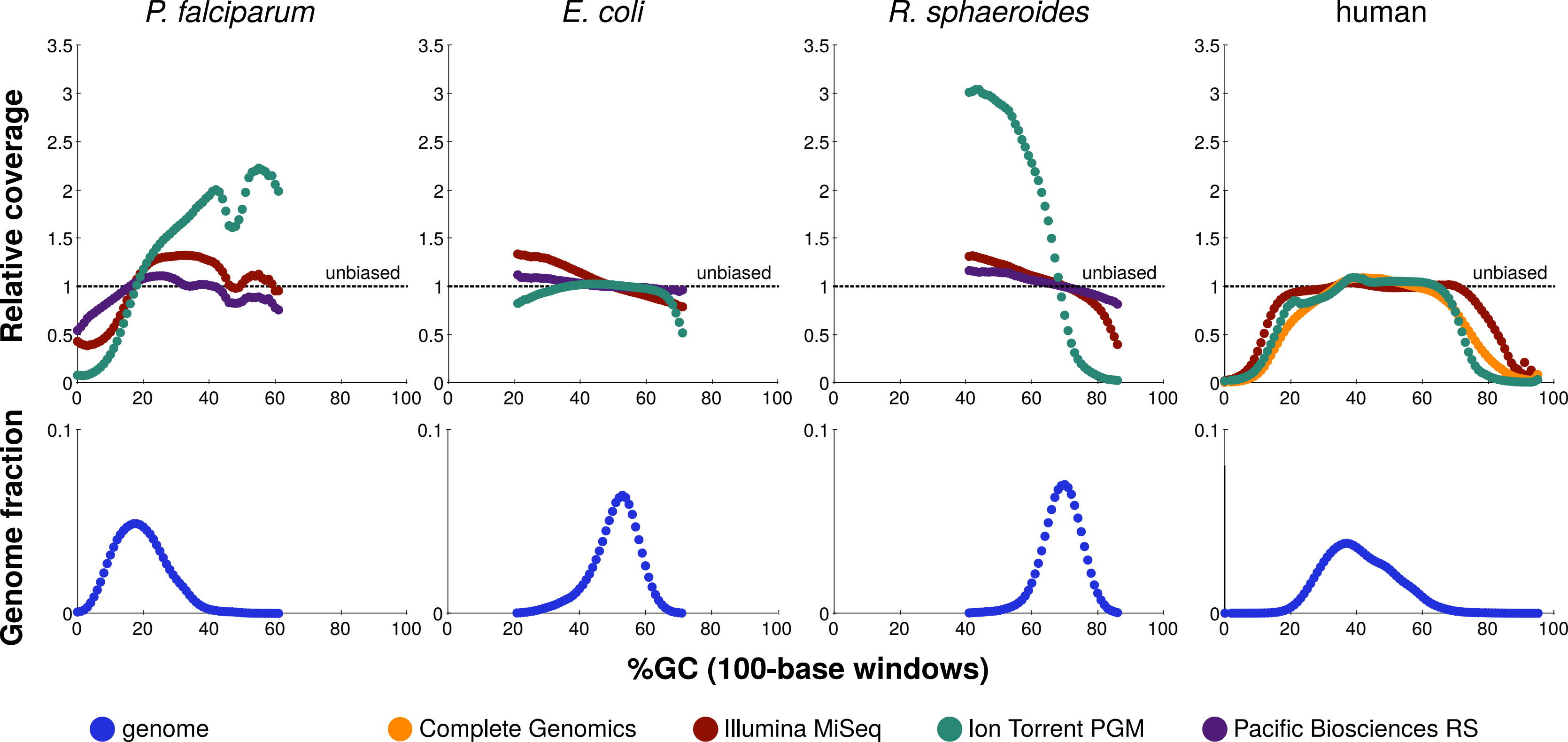

P. falciparum
E. coli
R. sphaeroides
human
Relative coverage
Genome fraction
unbiased
%GC (100-base windows)
genome
Complete Genomics
Illumina MiSeq
Ion Torrent PGM
Pacific Biosciences RS

Figure 2

Error rate biases in homopolymers of varying lengths and due to different local GC sequence content. (A) Top panels show the average error rates at homopolymers of different lengths per genome and platform. (B) Bottom panels show error rates across different GC sequence contents of 100-base windows.

This figure is aggregated and adapted from figures 4 and 5 in [13], according to the Creative Commons Attribution license CC-BY 2.0 (http://creativecommons.org/licenses/by/2.0/).

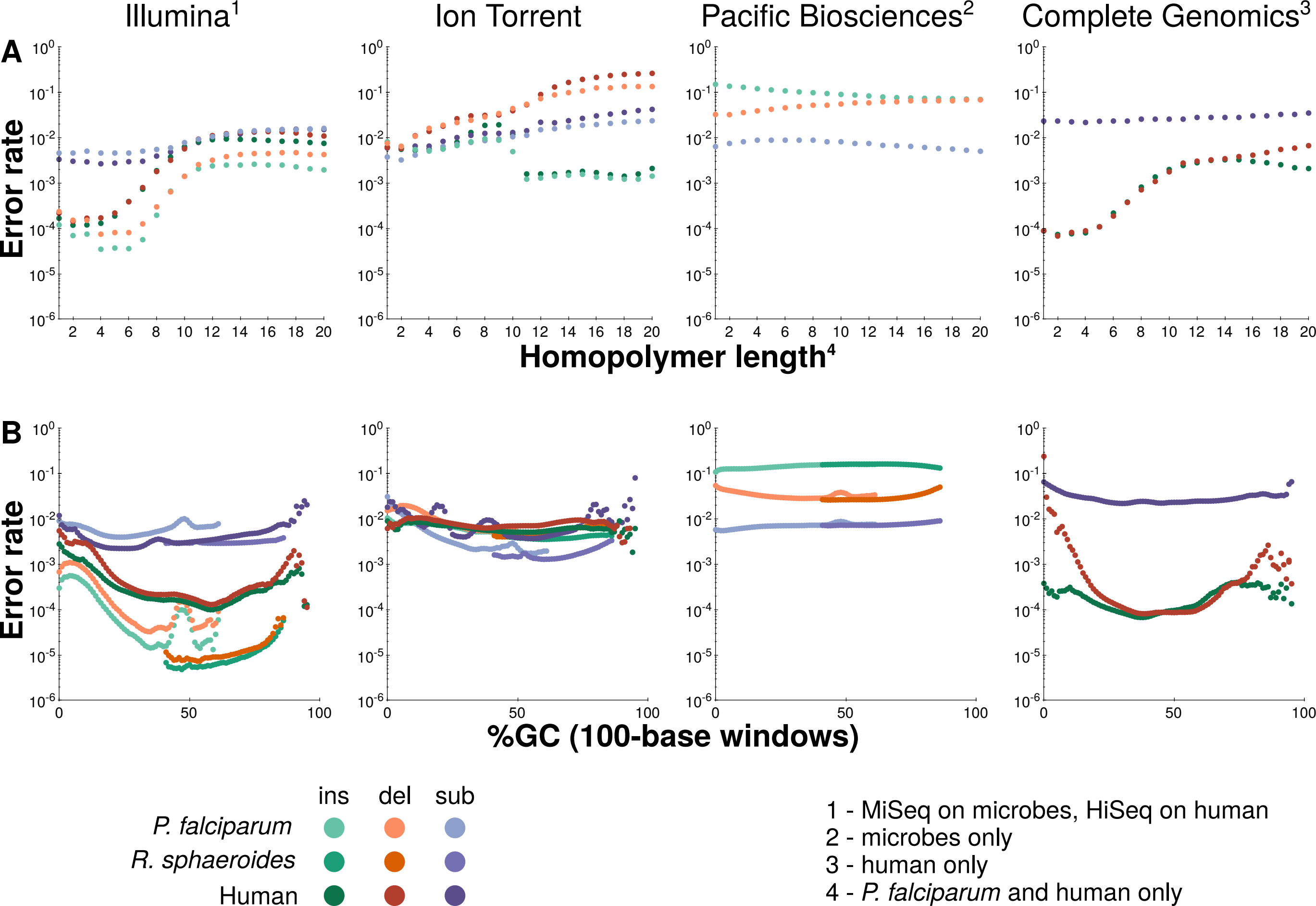

Illumina[1]
Ion Torrent
Pacific Biosciences[2]
Complete Genomics[3]
A
B
Error rate
Homopolymer length[4]
%GC (100-base windows)
ins
del
sub
P. falciparum
R. sphaeroides
Human
1 - MiSeq on microbes, HiSeq on human
2 - microbes only
3 - human only
4 - P. falciparum and human only

Figure 3

Overview how to generate a pileup from a read set depending on the error correction strategy. (A) If a good and close reference is known, reads can be mapped to it. Otherwise, one of the other approaches is necessary: (B) A multiple sequence alignment of reads can be formed from a pairwise alignments of all read pairs, of all reads with an overlap in an initial mapping to an available reference (dashed grey arrow from A to B), of all reads sharing part of a suffix (dashed grey arrow from F to B) or of all read pairs sharing a k-mer seed, identified by a table recording all reads that each k-mer occurs in (grey table and respective dashed grey arrows). Also, a simple recording of the count of all k-mers can be used to derive (C) a k-mer Spectrum or (D) a Hamming graph (Figure 4), and read suffixes of reads augmented with unique symbol ($\$x$) can be used to construct (E) a suffix trie (Figure 5) or (F) a suffix array (Figure 6).

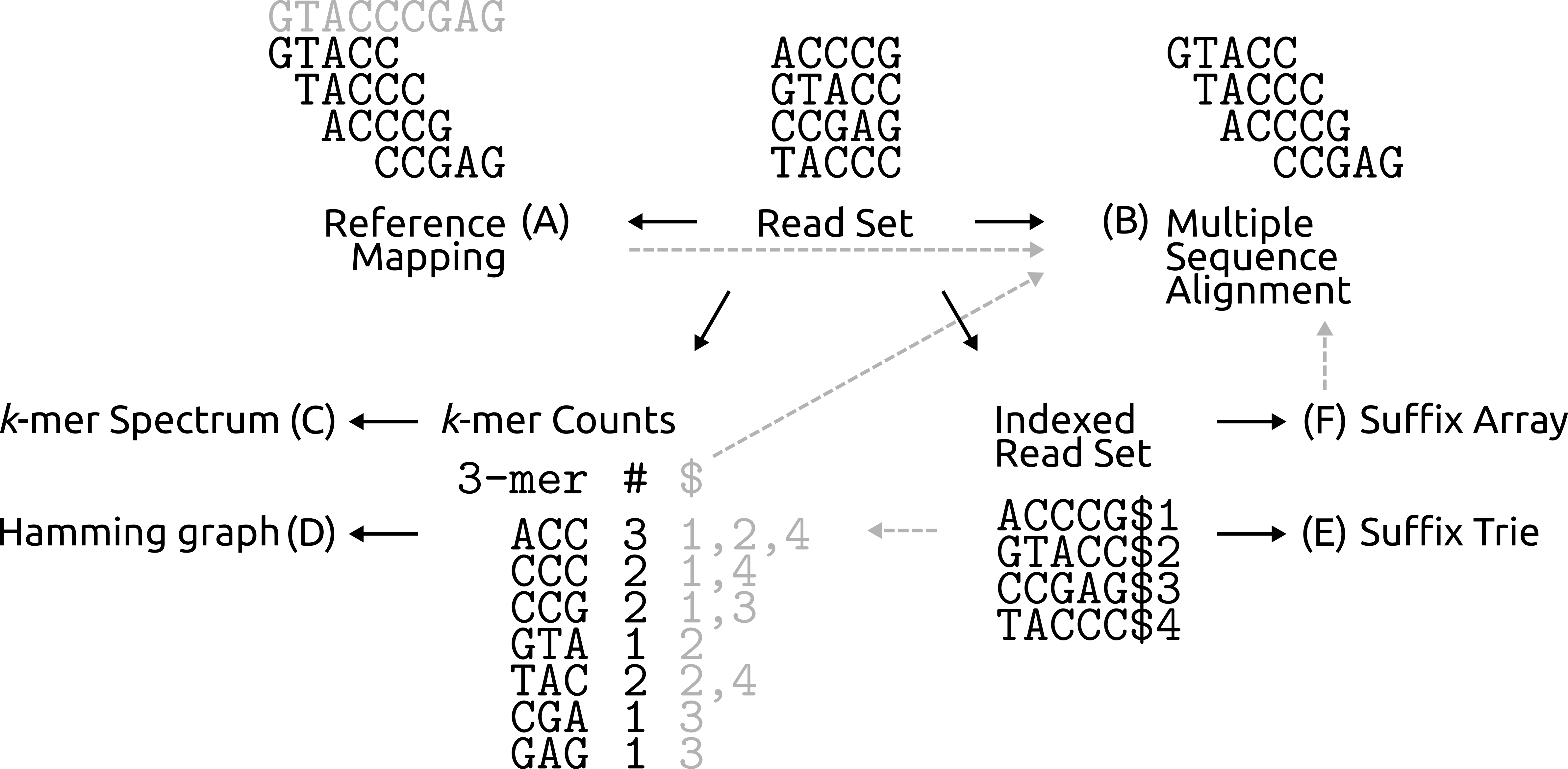
GTACCCGAG
GTACC
TACCC
ACCCG
CCGAG
Reference Mapping (A)
ACCCG
GTACC
CCGAG
TACCC
Read Set
GTACC
TACCC
ACCCG
CCGAG
(B) Multiple Sequence Alignment
k-mer Spectrum (C)
k-mer Counts
3-mer # $
ACC 3 1,2,4
CCC 2 1,4
CCG 2 1,3
GTA 1 2
TAC 2 2,4
CGA 1 3
GAG 1 3
Hamming graph (D)
Indexed Read Set
ACCCG$1
GTACC$2
CCGAG$3
TACCC$4
(F) Suffix Array
(E) Suffix Trie

Figure 4

Deriving a *k*-mer Spectrum or a Hamming graph from *k*-mer counts. Some error correction tools work directly with the *k*-mer frequencies as counted from the read set. Others set a minimum *k*-mer coverage (2 in this example, green) to consider a *k*-mer as correct (trusted *k*-mers, green counts) and then derive a (C) *k*-mer Spectrum of all trusted *k*-mers. In this simplified example, this step classifies *k*-mers from the end of the queried sequence as untrusted (the reference in Figure 3A could be considered the sequence queried by the four reads). By using a Bloom filter, space usage of the *k*-mer spectrum can be reduced. Another concept used in *k*-mer approaches is the Hamming graph, where nodes are *k*-mers from the read set and nodes are connected if the Hamming distance (i.e. the number of base substitutions between them; see also Section "Substitutions only vs. substitutions plus indels: Hamming vs. Levenshtein distance") is below a given threshold. In this simplified example, *k*-mers are too short and the Hamming graph therefore connects three correct *k*-mers. In a real setting, the *k*-mer length must be chosen with care (Section "Optimal k-mer length") and most connected components of the Hamming graph should contain only a single correct *k*-mer plus *k*-mers generated from the same sequence with errors.

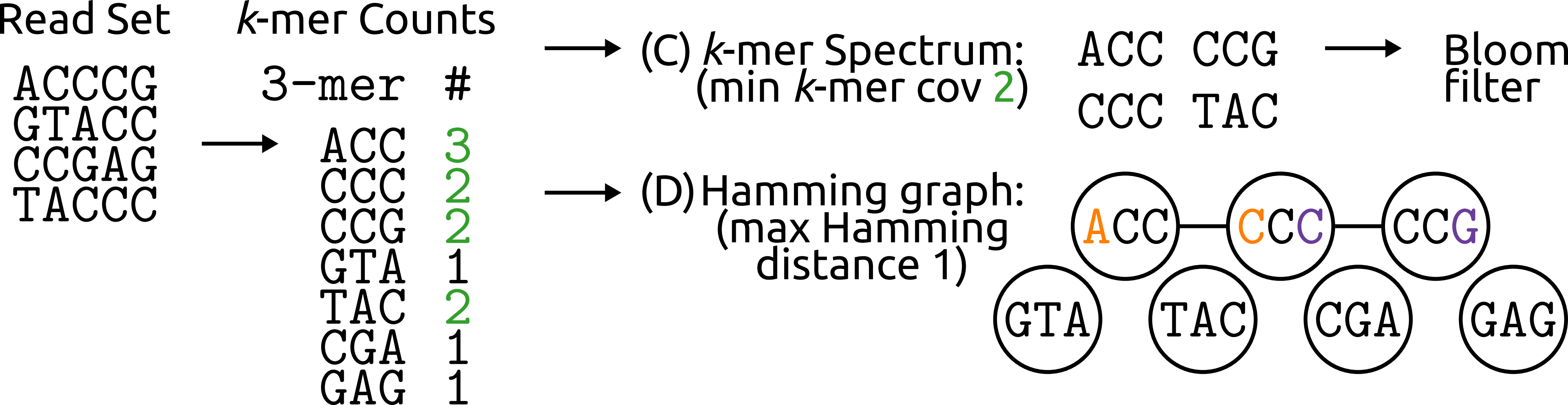

Read Set
ACCCG
GTACC
CCGAG
TACCC
k-mer Counts
3-mer #
ACC 3
CCC 2
CCG 2
GTA 1
TAC 2
CGA 1
GAG 1
(C) k-mer Spectrum: (min k-mer cov 2)
ACC CCG
CCC TAC
Bloom filter
(D) Hamming graph: (max Hamming distance 1)
ACC
CCC
CCG
GTA
TAC
CGA
GAG

Figure 5

A suffix trie is a tree of all suffixes from the indexed example read set. Every existing suffix can be spelled out by a path from the root node to one of the read indices, indicated by arrowheads and read numbers at corresponding nodes. Numbers at trie edges correspond to the number of suffixes passing through them, i.e. edge weights give the coverage of a sequence from the root down to the following node. E.g. the 2-mer “CC” occurs six times in the four example reads.

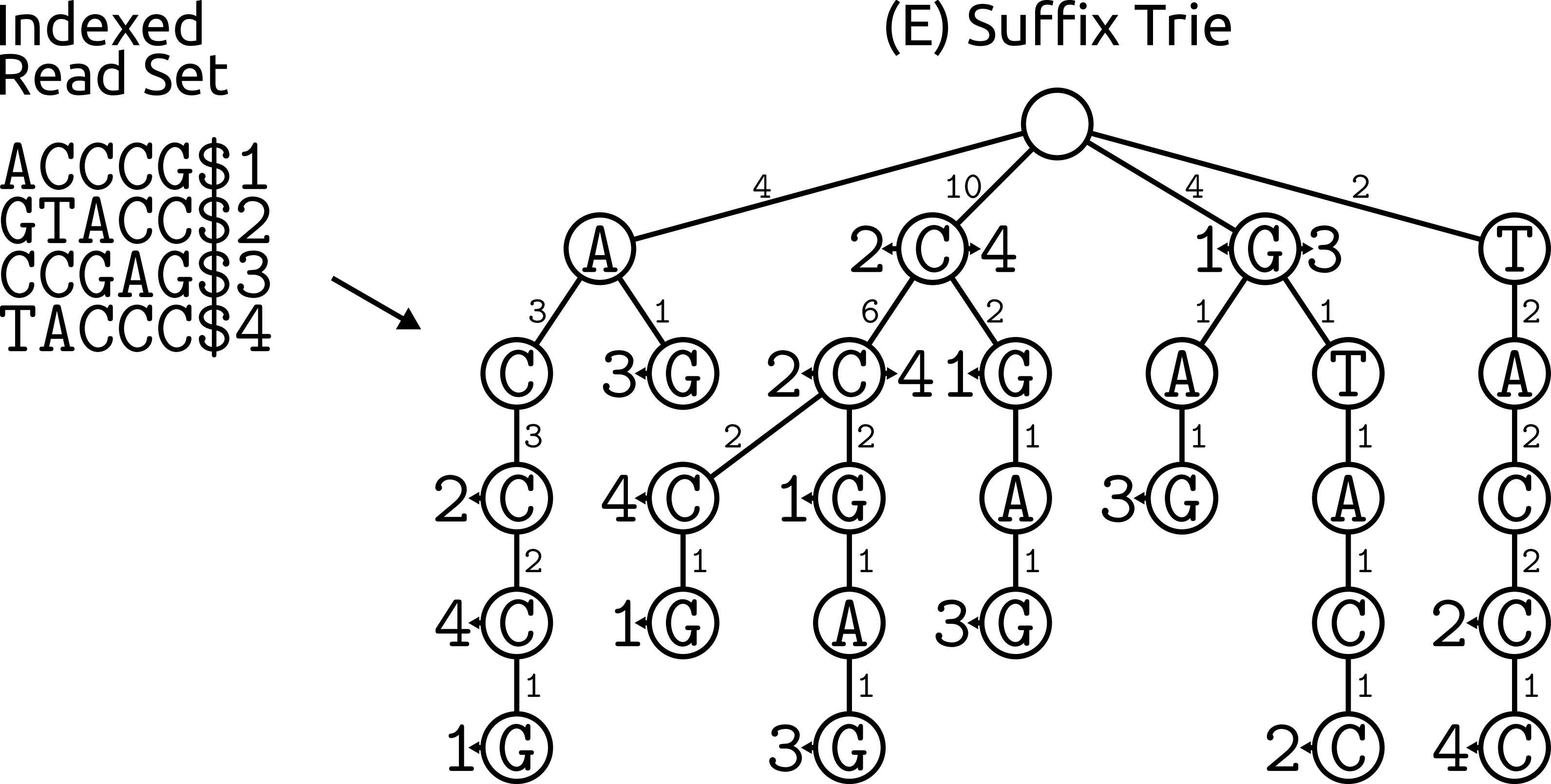
Indexed
Read Set
ACCCG$1
GTACC$2
CCGAG$3
TACCC$4
(E) Suffix Trie

Figure 6

Steps for deriving first a suffix array and then the Burrows Wheeler transform (BWT) and the FM index from the running example read set. Also given is an example for a string search using BWT and FM index, with the colors purple, red and green tracing corresponding indices and nucleotides. For suffix array construction, a unique termination symbol ($*x*) is appended to each read and reads are concatenated to a string *R* in lexicographical order of their termination symbol ($1<$2<$3<$4). All possible suffixes are formed and sorted unambiguously, as termination symbols have an order, as do the other symbols $<A<C<G<T. A suffix array entry at suffix array index *i* then corresponds to the position in string *R* at which the *i*-th (lexicographically) lowest suffix starts. The longest common prefix (LCP) of a suffix array entry and the preceding entry is then recorded and suffix array plus LCP already form an efficient data structure for determining string occurrence frequencies. The BWT enables further compression of the data. Here, an entry at BWT index *i* corresponds to the symbol before the *i*-th lowest suffix in *R*. Together with the FM index, it allows for linear time string searches (and thus also determination of the coverage of a string) in the whole read set. The FM index gives the number of occurrences of each of the symbols up to any index of the BWT and for each symbol counts all occurrences of all lexicographically lower symbols (e.g. 4+4+10=18 for symbol “G”, all counts in blue).

Indexed Read Set → Concatenated Reads → Suffixes → Sorted Suffixes → (F) Suffix Array → (G) Burrows Wheeler Transform → (H) Ferragina Manzini Index

Indexed Read Set

ACCCG$1
GTACC$2
CCGAG$3
TACCC$4

R, Full String	Position Index	Suffixes	Sorted Suffixes	Array Index	Array Entries	Longest Common Prefix	BWT Index	BWT Entries	FM Index	Occ $	Occ A	Occ C	Occ G	Occ T
A	0	ACCCG$1	$1	0	5	0	0	G	0	0	0	0	1	0
C	1	CCCG$1	$2	1	11	0	1	C	1	0	0	1	1	0
C	2	CCG$1	$3	2	17	0	2	G	2	0	0	1	2	0
C	3	CG$1	$4	3	23	0	3	C	3	0	0	2	2	0
G	4	G$1	ACC$2	4	8	0	4	T	4	0	0	2	2	1
$1	5	$1	ACCC$4	5	19	3	5	T	5	0	0	2	2	2
G	6	GTACC$2	ACCCG$1	6	0	4	6	$4	6	1	0	2	2	2
T	7	TACC$2	AG$3	7	15	1	7	G	7	1	0	2	3	2
A	8	ACC$2	C$2	8	10	0	8	C	8	1	0	3	3	2
C	9	CC$2	C$4	9	22	1	9	C	9	1	0	4	3	2
C	10	C$2	CC$2	10	9	1	10	A	10	1	1	4	3	2
$2	11	$2	CC$4	11	21	2	11	C	11	1	1	5	3	2
C	12	CCGAG$3	CCC$4	12	20	2	12	A	12	1	2	5	3	2
C	13	CGAG$3	CCCG$1	13	1	3	13	A	13	1	3	5	3	2
G	14	GAG$3	CCG$1	14	2	2	14	C	14	1	3	6	3	2
A	15	AG$3	CCGAG$3	15	12	3	15	$2	15	2	3	6	3	2
G	16	G$3	CG$1	16	3	1	16	C	16	2	3	7	3	2
$3	17	$3	CGAG$3	17	13	2	17	C	17	2	3	8	3	2
T	18	TACCC$4	G$1	18	4	0	18	C	18	2	3	9	3	2
A	19	ACCC$4	G$3	19	16	1	19	A	19	2	4	9	3	2
C	20	CCC$4	GAG$3	20	14	1	20	C	20	2	4	10	3	2
C	21	CC$4	GTACC$2	21	6	1	21	$1	21	3	4	10	3	2
C	22	C$4	TACC$2	22	7	0	22	G	22	3	4	10	4	2
$4	23	$4	TACCC$4	23	18	4	23	$3	23	4	4	10	4	2

Counts

Symbol	Count
$	0
A	4
C	8
G	18
T	22

```
Update formulas for iterative string search in suffix array:

f = C(x) + Occ(x,f-1)

l = C(x) + Occ(x,l) - 1

With:

    C(x) - count of occurrences of all symbols in R that are
           lexicographically lower than x

       f - first index of suffix array interval

       l - last index of suffix array interval

Occ(x,i) - count of occurrences of symbol x in all BWT
           positions up to i

       R - concatenation of all reads

       Q - query string (of symbols from alphabet)

       x - symbol from alphabet
```

```
Example Search for Q "TAC" in read concatenation R:

   (i) Initialize [f,l]
        Suffix array interval corresponding to suffixes
        starting with last letter "C":
         f = C(C) = 8
         l = C(G) - 1 = 17
         -> [8,17]

  (ii) 1st interval update
        To suffix array interval starting with "AC":
         f = C(A) + Occ(A,7) = 4 + 0 = 4
         l = C(A) + Occ(A,17) - 1 = 4 + 3 - 1 = 6
         -> [4,6]

 (iii) 2nd interval update
        To suffix array interval starting with "TAC":
         f = C(T) + Occ(T,3) = 22 + 0 = 22
         l = C(T) + Occ(T,6) - 1 = 22 + 2 - 1 = 23
         -> [22,23]

  (iv) Positions of Q in R
         Entries in suffix array at indices [22,23]
         -> R[7] and R[18]
```

Figure 7

k-mer coverage histogram with a model fit. The histogram in this plot from the Quake paper [70] gives a nice example of an empirical *k*-mer coverage distribution. The density tells us which proportion of all existing *k*-mers in the dataset has a particular coverage. The solid line gives the Quake model fit. The first peak of the distribution is formed by very low coverage error *k*-mers and is usually modelled by a Poisson or a Gamma distribution. The second peak results from the majority of correct *k*-mers and is usually modelled by a Poisson or a Gaussian distribution. Between these two peaks, a clear local minimum can provide a *k*-mer trust coverage cut-off. The heavy tail of higher multiplicity *k*-mers is the result of *k*-mers from sequence repeats. Quake draws the *k*-mers' sequence copy numbers from a Zeta distribution and then projects these *k*-mers into the coverage range of the correct *k*-mers.

Adapted by font change and label addition from [70], according to the Creative Commons Attribution license CC-BY 2.0 (http://creativecommons.org/licenses/by/2.0/).

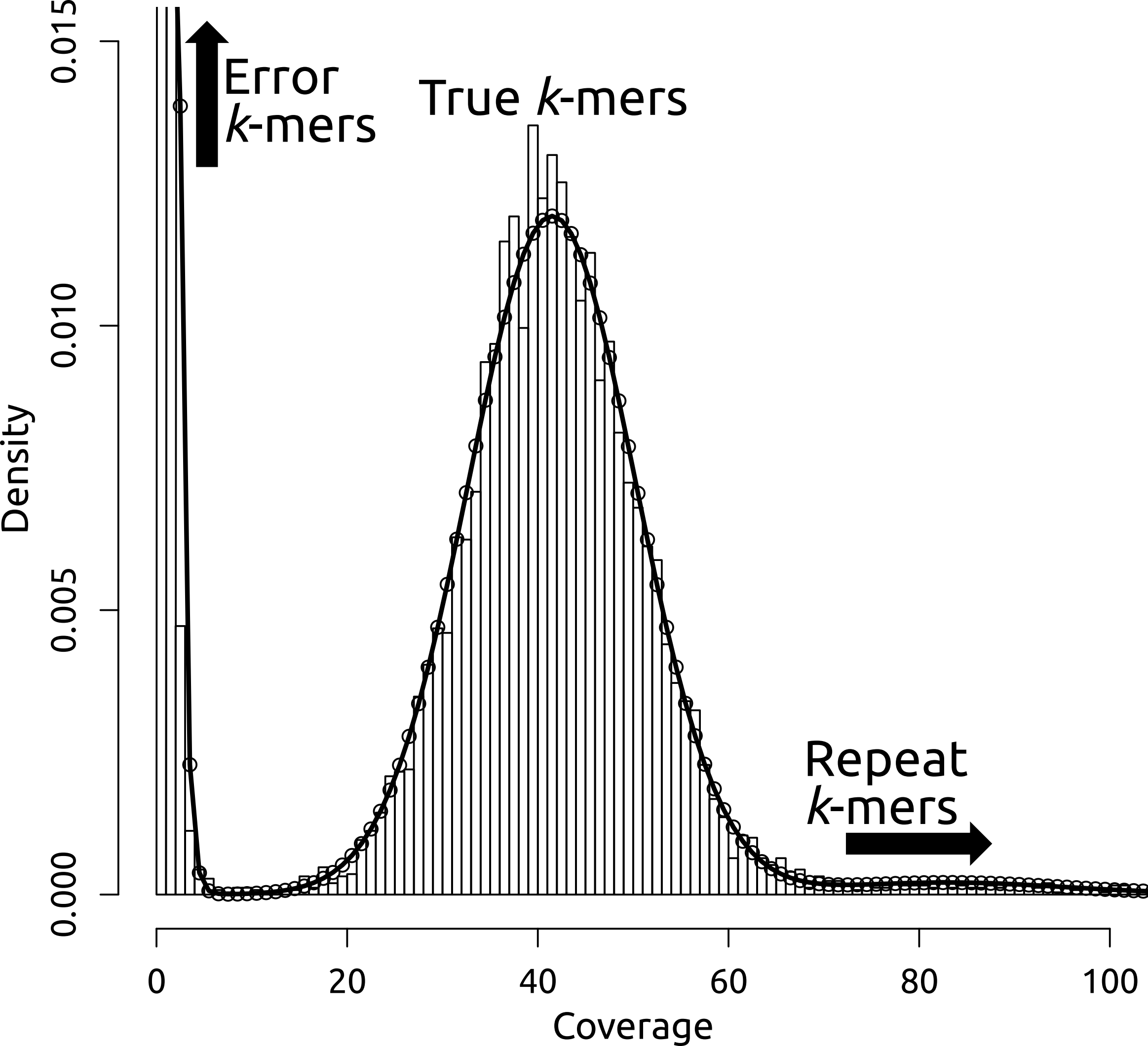

Error
k-mers
True k-mers
Repeat
k-mers
0.015
0.010
0.005
0.000
Density
0
20
40
60
80
100
Coverage

Figure 8

Example of a weighted de Bruijn graph from the example read set. The read set is augmented to include reads that show variation compared to the example read set in the earlier figures: while the Ts (red and orange) could be substitution errors and the G (purple) could be an insertion error, all three could also be reads covering alternative alleles of the same sequence locus or slightly different repeats at other sequence loci. The new reads create graph structures that are commonly removed in graph pruning (and thus correction) steps: (i) the orange T creates a bulge, a cycle in the graph that can not be traversed by a single path, as edges of the cycle go in opposing directions (e.g. edges ACC and TCC both ending at node CC); (ii) the red T creates a tip, a short dead end of the graph; (iii) the purple G creates a whirl, a cycle in the graph with a possible path going around the whole cycle (i.e. all edges go in the same direction). A repeat graph would have small (and assumingly erroneous) whirls, bulges and tips removed and repeats collapsed, but care must be taken to not remove genuine variation.

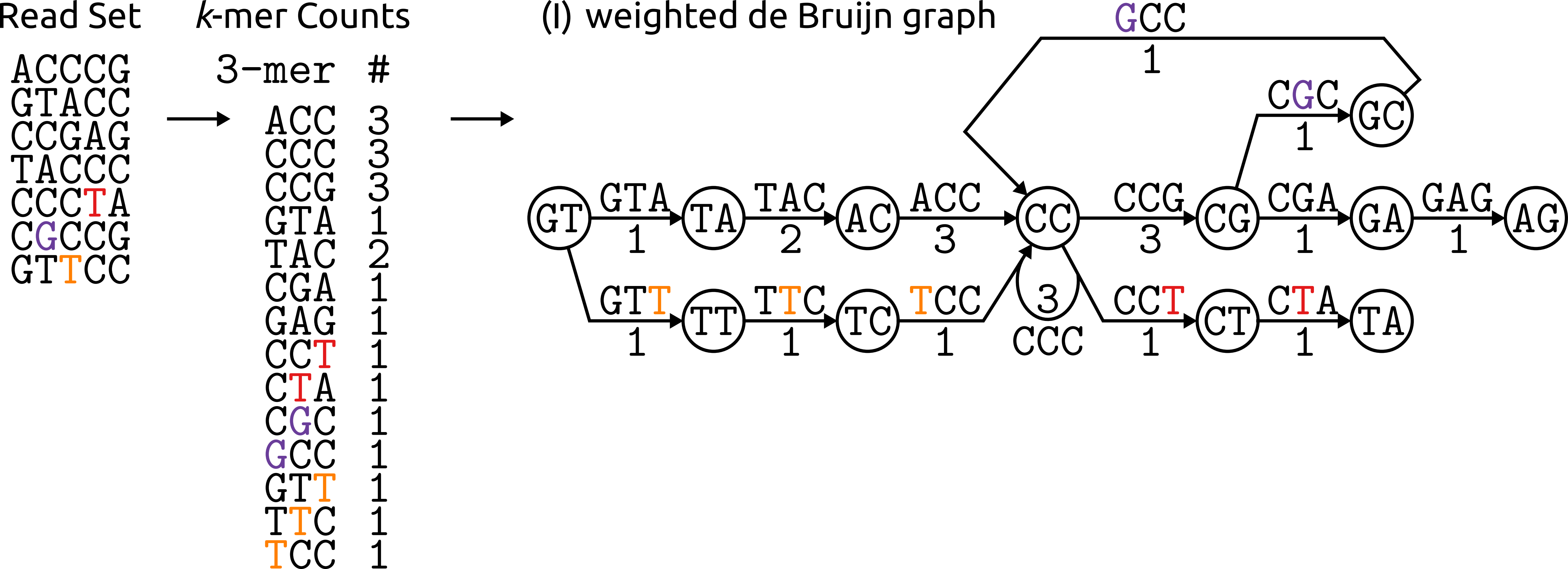
Read Set
ACCCG
GTACC
CCGAG
TACCC
CCCTA
CGCCG
GTTCC
k-mer Counts
3-mer #
ACC 3
CCC 3
CCG 3
GTA 1
TAC 2
CGA 1
GAG 1
CCT 1
CTA 1
CGC 1
GCC 1
GTT 1
TTC 1
TCC 1
(I) weighted de Bruijn graph
GT
TA
AC
CC
CG
GA
AG
GC
TT
TC
CT
GTA 1
TAC 2
ACC 3
CCG 3
CGA 1
GAG 1
GCC 1
CGC 1
GTT 1
TTC 1
TCC 1
3 CCC
CCT 1
CTA 1

Figure 9

Example of a multiple sequence alignment (MSA) of a read set, demonstrating consistent mismatches (green nucleotides) in comparison to isolated or low-frequency mismatches and indels (red nucleotides and dashes). The consistency of mismatches can be tested through their linkage within reads (four in this example) and are called defined nucleotide positions (DNPs) in the first tool that used such information, MisEd [86,87]. However, genuine polymorphisms can only be told from sequencing errors if multiple of the linked variant sites are within the range covered by the average read (pair).

```
CGTAGCGTGA
 GTAGAGTGAC
 GTAGCGAGAC
  TAGCGTGACC
  TAGAGTGACGC
   AGCGTGACCC
     CGGGACCGC-G
     CGTGACCCC-G
      GTGACCCCCG
       TGACCGC-GTCC
        GACCCC-GGCC
         ACCGC-GTCCG
         ACCCC-GGC-GG
          CCGC-GTCCGG
            CC-GGCCGGTA
            GC-GTCCGGTA
             C-GGCCGGTAC
               GTCCGGAACT
                 CCGGTACTAT
                  CGGTACTAGC
```
